# Dietary intake and cardiometabolic risk factors among Venezuelan adults: a nationally representative analysis

**DOI:** 10.1186/s40795-020-00362-7

**Published:** 2020-10-16

**Authors:** Dina Goodman, Juan P. González-Rivas, Lindsay M. Jaacks, Maritza Duran, María Inés Marulanda, Eunice Ugel, Josiemer Mattei, Jorge E. Chavarro, Ramfis Nieto-Martinez

**Affiliations:** 1 Department of Global Health and Population, Harvard TH Chan School of Public Health, Boston, MA USA; 2 International Clinical Research Center (ICRC), St. Ann’s University Hospital, Brno, Czech Republic; 3 Foundation for Clinic, Public Health, and Epidemiology Research of Venezuela (FISPEVEN INC), Caracas, Venezuela; 4 Endocrine Associates of Florida, Research Department, Orlando, Florida USA; 5 Public Health Research Unit, Department of Social and Preventive Medicine, School of Medicine, Universidad Centro-Occidental “Lisandro Alvarado”, Barquisimeto, Venezuela; 6 Department of Nutrition, Harvard TH Chan School of Public Health, Boston, MA 02115 USA; 7 LifeDoc Health, Memphis, TN USA

**Keywords:** Nutrition transition, Western diet, Obesity, Venezuela, Latin America

## Abstract

**Background:**

Increasing trends in global obesity have been attributed to a nutrition transition where healthy foods are replaced by ultra-processed foods. It remains unknown if this nutrition transition has occurred in Venezuela, a country undergoing a socio-political crisis with widespread food shortages.

**Methods:**

We described dietary intake of Venezuelans from a nationally representative study conducted between 2014 and 2017. We conducted a cross-sectional analysis of dietary, sociodemographic, and clinical data from Venezuelans ≥20 years of age (*n* = 3420). Dietary intake was assessed using a semi-quantitative food frequency questionnaire. Standardized clinical and anthropometric measurements estimated obesity, type 2 diabetes, and hypertension. A Dietary Diversity Score (DDS) was calculated using an amended Minimum Dietary Diversity for Women score where the range was 0 to 8 food groups, with 8 being the most diverse. Analyses accounted for complex survey design by estimating weighted frequencies of dietary intake and DDS across sociodemographic and cardiometabolic risk-based subgroups.

**Results:**

The prevalence of obesity was 24.6% (95% CI: 21.6–27.7), type 2 diabetes was 13.3% (11.2–15.7), and hypertension was 30.8% (27.7–34.0). Western foods were consumed infrequently. Most frequently consumed foods included coffee, arepas (a salted corn flour cake), and cheese. Mean DDS was 2.3 food groups (Range: 0–8, Standard Error: 0.07) and this score did not vary among subgroups. Men, younger individuals, and those with higher socioeconomic status were more likely to consume red meat and soft drinks once or more weekly. Women and those with higher socioeconomic status were more likely to consume vegetables and cheese once or more daily. Participants with obesity, type 2 diabetes, and hypertension had lower daily intake of red meat and arepas compared to participants without these risk factors.

**Conclusions:**

Despite high prevalence of cardiometabolic risk factors, adults in Venezuela have not gone through a nutrition transition similar to that observed elsewhere in Latin America. Dietary diversity is low and widely consumed food groups that are considered unhealthy are part of the traditional diet. Future studies are needed in Venezuela using more comprehensive measurements of dietary intake to understand the effect of the socio-political crisis on dietary patterns and cardiometabolic risk factors.

## Background

The relationship between suboptimal dietary intake and noncommunicable diseases (NCDs) is well-established. In 2017, 11 million deaths and 255 million disability-adjusted life years (DALYs) were attributable to dietary risk factors, a considerable increase from an estimated 8 million deaths and 184 DALYs in 1990 [1]. Concurrently, the global prevalence of obesity, as well as related cardiometabolic diseases such as type 2 diabetes (T2D) and hypertension, increased substantially over the past 40 years [2].

The nutrition transition [3, 4] describes the process where a high prevalence of undernutrition is replaced by overnutrition through large changes in dietary intake and physical activity patterns, resulting in a diet primarily consisting of westernized, ultra-processed foods [5, 6]. Though long believed to only affect individuals with high socioeconomic status, the obesity epidemic has increasingly spread to lower socioeconomic groups [7]. Several Latin American countries, including Argentina [7], Mexico [8, 9], and Brazil [10, 11] have demonstrated this shifting burden across socioeconomic groups [7].

Venezuela is a particularly salient case study as it is currently undergoing an economic and socio-political crisis that has led to widespread food shortages and malnutrition [12], factors that may reverse the nutrition transition. Prior to the crisis, the burdens of T2D, hypertension, and obesity were documented to be increasing over time, particularly in urban areas [13, 14]. However, few studies have looked at recent dietary intake in Venezuela [15, 16] and most of these have been limited to convenience samples rather than nationally representative data. Furthermore, NCD burden has been especially hard to quantify over the past few years, as the Venezuelan government has stopped publishing national statistics since 2016 [17].

This paper aims to describe the relationship between dietary intake and obesity, hypertension, and T2D, using a nationally representative dataset from EVESCAM (Estudio Venezolano de Salud Cardio-Metabólica). EVESCAM, conducted between 2014 and 2017, was the first nationally-representative study in Venezuela on risk factors for cardiometabolic disease, with data on NCDs including T2D, obesity, and hypertension; and lifestyle risk factors for these diseases [18].

## Methods

### Study population

Data are from EVESCAM, a population-based, cluster-sampled study. Details of the study design and sampling strategy have been published elsewhere [18]. Briefly, between July 2014 and January 2017, 4454 study participants were enrolled through a multi-stage stratified sampling method, using parish as the primary sampling unit. As such, these data are representative at the national level. Enrollment occurred at the household-level, where all members aged ≥20 years were invited to participate. Exclusion criteria included pregnancy, inability to stand or communicate, or refusal to participate.

### Dietary intake assessment

Dietary intake was ascertained using a semi-quantitative food frequency questionnaire that was developed through a working group of Venezuelan nutrition experts and hosted by the EVESCAM principal investigators. The questionnaire (Additional file 1) asked participants to list frequency of consumption and portion size for 33 food groups, based on show cards used to help estimate portion sizes accurately (Additional file 2). Responses were categorized by frequency: daily (1 portion, 2–4 portions, or ≥ 5 portions per day), weekly (1 portion, 2–4 portions, or ≥ 5 portions per week), or monthly (0 portion, 1-3 portions). Responses were converted to frequency of daily intake using the median value of each category (e.g. 2–4 portions per week was recoded as 3 times per week or 0.43 times per day; 1–3 times per month was recoded as 2 times per month or 0.07 times per day). Water, sugar, and alcohol were excluded from this analysis.

A Dietary Diversity Score (DDS) was calculated to indicate the number of different food categories that participants reported consuming. This score was calculated based on Minimum Dietary Diversity-Women (MDD-W) developed by the Food and Agriculture Organization of the United Nations [19]. Based on the food groups collected in EVESCAM, the DDS used eight food categories rather than ten in MDD-W. This analysis categorized food groups as: 1) grains, white roots and tubers, plantains; 2) pulses; 3) Nuts and seeds; 4) Dairy; 5) Meat-based foods: red meat, poultry and fish; 6) Eggs; 7) Fruits 8) Vegetables. Food groups included in each category are listed in Table 4 (Appendix 1). Each food category was given a score of zero if consumed weekly or less or one if at least one portion was consumed daily, and then a final score was created by summing all eight categories. Thus, a score of eight represents the most diverse diet and zero the least diverse diet.

### Covariate assessment

Sociodemographic variables included sex, age, and socioeconomic status (SES). SES was calculated using a version of the Graffar Scale modified for Venezuela [20], which pools income, profession, educational level, and housing conditions into a composite score. Each variable is rated independently from one to five, with one being the highest level of SES. A final score sums the independent ratings and classifies participants’ SES as high, medium-high, medium, medium-low, and low [20]. Few participants were in the highest quintile (1.3%) so the two highest categories were merged. Data on sex, age, and SES were missing for < 5% of participants.

Weight was measured with the lightest possible clothes, without shoes, using a calibrated scale (Tanita UM-081®, Japan). Height was measured using a portable stadiometer (Seca 206® Seca GmbH & Co., Hamburg, Germany). Body mass index (BMI) was defined as weight (measured in kilograms) divided by height (measured in meters) squared, and classified as underweight (< 18.5 kg/m^2^), normal weight (18.5 to < 25.0 kg/m^2^), overweight (25.0 to < 30.0 kg/m^2^), or obesity (≥30.0 kg/m^2^) [21].

Blood pressure was measured twice, in five-minute intervals, in the right arm using a validated oscillometric sphygmomanometer (Omron HEM-705C Pint® Omron Healthcare CO., Kyoto/Japan) [22]. Participants were seated and rested their arm at heart level. Hypertension was defined as having a systolic blood pressure ≥ 140 mmHg, diastolic blood pressure ≥ 90 mmHg, or self-report of antihypertensive medication use [23].

Blood glucose measurements included fasting blood glucose and a 2-h oral glucose tolerance test (OGTT) using a 300-ml test solution containing 75 g anhydrous glucose. Diabetes was defined as either: fasting plasma glucose ≥126 mg/dL, 2-h after 75 g oral glucose tolerance test ≥200 mg/dL, or self-report of diabetes medication use [24]. The mean age of diagnosis was 50.6 years and 7% of participants were on insulin only, so we refer to all participants as having T2D. Sensitivity analyses excluding one participant diagnosed at < 21 years of age and on insulin only did not change results (data not shown).

### Statistical analysis

Of 4454 participants screened, 3420 were enrolled in EVESCAM and included in the present analysis. As food group consumption was not always normally distributed, reporting only mean daily consumption would have been statistically inaccurate. For this reason, medians and interquartile ranges (IQR) were graphically represented using boxplots, one set of boxplots showing median consumption of Western foods and another for traditional foods. Western foods (eight total) included white bread, red meat, cookies, cake, soft drinks, fast food, french fries, and burgers [4]. Traditional foods (17 total) were determined based on previous studies categorizing Venezuelan dietary patterns [13], and included arepas (a salted corn flour cake), coffee, cheese, white rice, vegetables, fruits, fruit juice, empanadas, oats, legumes, fish, poultry, eggs, plantains, potatoes, pasta, and soup. Cereal, cachapa, and nuts were not included in the boxplots as they were consumed monthly or less by over 75% of participants.

**Fig. 1:**
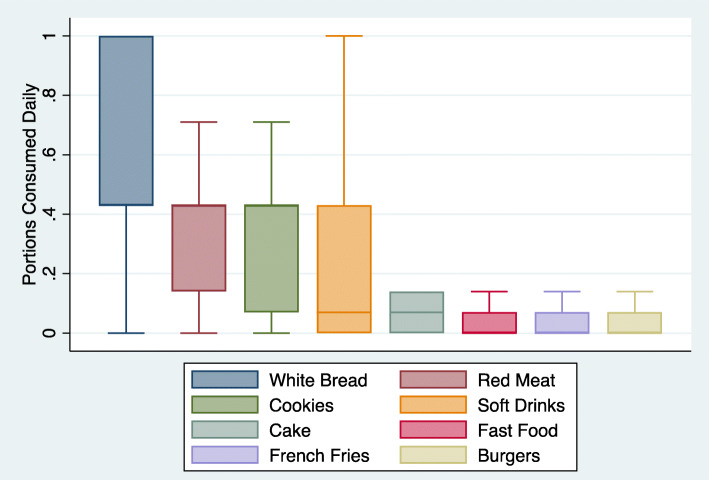
Boxplot of consumption of Western foods among Venezuelan adults, 2014–2017. The values displayed here are median and interquartile range. This figure excludes outliers. Food groups are displayed as portions consumed daily

**Fig. 2:**
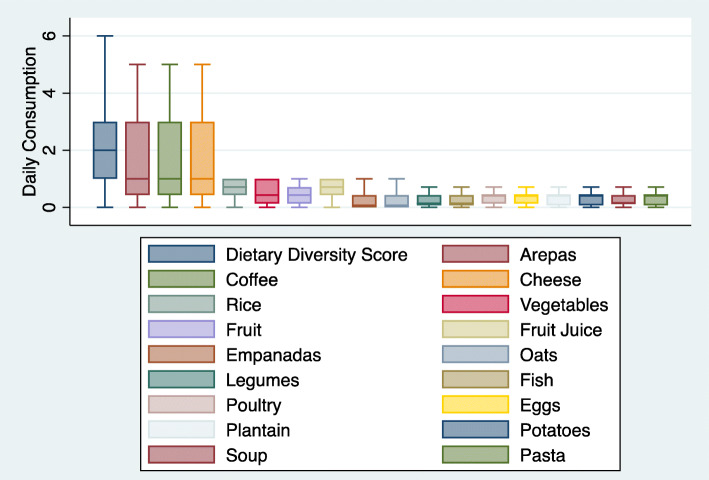
Boxplots of consumption of traditional food groups and distribution of Dietary Diversity Score among Venezuelan adults, 2014–2017. The values displayed here are medians and interquartile ranges. This figure excludes outliers. Dietary diversity score was calculated as an amended minimum dietary diversity-women (MDD-W) score, where each food category was given a score of 0 if consumed weekly or less or 1 if at least one portion was consumed daily, and then a final score was created by summing all eight categories. Food groups included in each category are listed in Appendix 1 in Table 4. Individual food groups are displayed as portions consumed daily

Bivariate analyses accounting for complex survey design were conducted to estimate dietary intakes by sociodemographic characteristics, including age, sex, and SES, and clinical subgroups (i.e. body mass, hypertension, and T2D status) using Pearson chi-squared tests. Differences between mean DDS by subgroup were evaluated using Somers’ D, a rank-sum test appropriate for weighted data [25]. This test also calculates jackknife standard error, adjusted for the primary sampling unit. Food groups were included in the bivariate analysis if they had a skewed distribution (Figs. 1 and 2) to understand if their consumption might differ by subgroups and if they were consumed weekly or daily by > 25% of participants.

All analyses were performed in Stata 16.0 (College Station, Texas, USA).

## Results

Weighted characteristics of all participants, and by sex, are summarized in Table 1. Briefly, participants (*n* = 3420) were between 20 and 96 years of age, with a mean age of 41.2 years (Standard Deviation (SD): 0.67) and 52.3% were female. The majority of participants lived in urban areas; only 19.3% of participants lived in rural areas. Most participants had BMI classified as overweight (34.5%; 95% Confidence Interval (CI): 31.8–37.4) or normal weight (36.6%; 32.5–40.7). Weighted prevalence of obesity was 24.6% (95% CI: 21.6–27.7), T2D 13.3% (11.2–15.7), and hypertension 30.8% (27.7–34.0). Underweight (4.3%; 95% CI: 3.2–5.8), extreme poverty (5.6%; 3.8–8.3), and high SES (2.0%; 0.9–4.4) were uncommon in the sample. Compared to females, there were significantly more male participants with T2D (15.9% of males vs 10.9% of females, *p*-value< 0.001) and hypertension (32.8% of males vs 28.9% of females, p-value = 0.055).

**Table 1:** Sociodemographic and clinical characteristics of adults*

	Weighted % (95%CI)			***P***-value^**1**^
	Total (n^2^ = 3402)	Male (n^2^ = 1052)	Female(n^2^ = 2348)	
Overall		47.7 (45.2–50.3)	52.3 (49.7–54.8)	
**Socioeconomic Status**^3^				0.320
High & Medium-High	21.2 (17.1–25.9)	22.3 (17.3–28.3)	20.1 (16.4–24.5)	
Medium	31.0 (27.8–34.3)	30.7 (26.6–35.2)	31.2 (28.1–34.5)	
Relative Poverty	42.2 (37.4–47.1)	40.7 (35–46.7)	43.6 (38.8–48.5)	
Extreme poverty	5.6 (3.8–8.4)	5.1 (3.4–7.5)	5.1 (3.4–7.5)	
**Age Category**				0.034
20–34	40.3 (36.0–44.6)	38.7 (32.9–44.9)	41.7 (37.3–46.3)	
35–44	21.5 (19.1–24.1)	20.9 (17.3–25.1)	22.1 (20.1–24.2)	
45–54	17.0 (15.6–18.6)	17.4 (15.1–19.9)	16.7 (15.1–18.5)	
55–64	11.4 (10.1–12.8)	11.1 (9.6–12.9)	11.6 (10.1–13.4)	
≥ 65	9.8 (8.0–11.8)	11.8 (9.7–14.4)	7.9 (6.3–9.8)	
**Locality**				0.047
Rural	19.3 (9.1–36.4)	17.7 (8.4–33.6)	20.8 (9.7–39.0)	
Urban	80.7 (63.6–90.9)	82.3 (66.4–91.6)	79.2 (61.0–90.3)	
**BMI Category**^4^				0.008
Underweight	4.3 (3.2–5.8)	3.5 (2.3–5.3)	5.1 (3.6–7.2)	
Normal Weight	36.6 (32.5–40.7)	36.2 (30.3–42.4)	36.9 (33.7–40.2)	
Overweight	34.5 (31.8–37.4)	38.2 (34.3–42.2)	31.2 (28.5–34.2)	
Obesity	24.6 (21.7–27.7)	22.2 (18.2–26.8)	26.7 (24.2–29.4)	
**Type 2 Diabetes**^**5**^	13.3 (11.2–15.7)	15.9 (13.2–19.0)	10.9 (8.9–13.3)	< 0.001
**Hypertension**^6^	30.8 (27.7–34.0)	32.8 (29.2–37.2)	28.9 (25.7–32.2)	0.055

^*^Estimates are weighted to be representative of Venezuelan adults over 20 years of age

^1^ *P*-values calculated using Pearson’s chi-square

^2^ n = unweighted sample size

^3^ SES was calculated using a version of the Graffar Scale modified for Venezuela [19], which combines income, profession, educational level, and housing conditions into a composite score

^4 ^BMI was defined as weight (measured in kilograms) divided by height (measured in meters) squared, and classified as underweight (< 18.5 kg/m^2^), normal weight (18.5 to < 25.0 kg/m^2^), overweight (25.0 to < 30.0 kg/m^2^), or obesity (≥30.0 kg/m^2^) [21]

^5 ^Type 2 diabetes was defined by either: fasting plasma glucose was ≥126 mg/dL, 2-h after 75 g oral glucose tolerance test ≥200 mg/dL, or self-report of diabetes medications [24]

^6 ^Hypertension was defined as having a systolic blood pressure ≥ 140 mmHg, diastolic blood pressure ≥ 90 mmHg, or self-report of antihypertensive medication use [23]

### Consumption of Western & Traditional Foods

White bread had the most frequent and variable consumption of the Western food groups (Fig. 1), with a median consumption of approximately three portions weekly (Median: 0.43, IQR: 0.43–1 portions daily). Red meat and cookies also had median consumption of three portions weekly but lower IQRs than white bread (red meat: 0.14–0.43 portions daily; cookies: 0.07–0.43 portions daily). Soft drinks had variable consumption patterns, despite low median consumption of 0.07 portions daily or approximately two portions monthly. Consumption of cake, french fries, burgers, and fast food was infrequent, with over 75% participants reporting consuming these food groups monthly or less.

Across all participants, median DDS was 2.00 (IQR: 1–3). The most commonly consumed traditional foods were arepas, coffee, and cheese, each with a median consumption of one time daily (IQR: 0.43–3 portions daily) (Fig. 2).

### Distribution of consumption by sociodemographic subgroups

Weighted bivariate analyses were conducted for DDS, white bread, red meat, cookies, soft drinks, arepas, coffee, cheese, vegetables, and fruits (Tables 2 and 3). As shown in Table 2, mean DDS was 2.28 (SE: 0.07) and did not vary by any sociodemographic subgroups.

**Table 2:** Percent of Venezuelan adults consuming food groups by sociodemographic subgroups*

	Total	Sex			SES^1^					Age						Locality		
		Male	Female	P-value^**2**^	High & Medium- High	Medium	Relative Poverty	Extreme Poverty	P-value^**2**^	20–34	35–44	45–54	55–64	≥65y	P-value^**2**^	Rural	Urban	P-value^**2**^
**n**^**3**^	3402	1057	2345		656	1017	1441	241		682	577	700	744	699		554	2848	
**DDS**^**4**^				0.131					0.734						0.703			0.405
*Mean (SE)*	2.28 (0.07)	2.21 (0.08)	2.34 (0.07)		2.27 (0.09)	2.30 (0.79)	2.29 (0.07)	2.37 (0.14)		2.28 (0.10)	2.33 (0.08)	2.23 (0.09)	2.31 (0.06)	2.19 (0.09)		2.11 (0.20)	2.32 (0.06)	
**White Bread**				0.005					0.126						0.293			0.274
*Monthly*	10%	7%	12%		7%	9%	11%	12%		9%	10%	8%	11%	12%		10%	9%	
*Weekly*	58%	58%	59%		56%	60%	59%	54%		61%	60%	58%	54%	52%		64%	57%	
*Daily*	32%	35%	29%		37%	31%	30%	34%		30%	31%	33%	36%	37%		26%	33%	
**Red Meat**				0.025					< 0.001						< 0.001			0.003
*Monthly*	19%	16%	21%		14%	13%	23%	36%		13%	17%	19%	27%	35%		28%	16%	
*Weekly*	74%	77%	72%		80%	80%	69%	60%		78%	76%	75%	70%	61%		67%	76%	
*Daily*	7%	7%	7%		6%	7%	8%	4%		9%	7%	6%	3%	4%		5%	8%	
**Cookies**				0.0154					0.963						0.016			0.298
*Monthly*	28%	31%	25%		28%	28%	28%	28%		26%	26%	30%	30%	34%		25%	29%	
*Weekly*	53%	51%	55%		52%	54%	53%	51%		52%	57%	54%	51%	51%		60%	51%	
*Daily*	19%	18%	20%		20%	18%	19%	21%		22%	17%	16%	19%	15%		14%	20%	
**Soft drinks**				0.002					0.081						< 0.001			0.460
*Monthly*	50%	46%	54%		47%	51%	50%	61%		37%	48%	57%	68%	73%		53%	49%	
*Weekly*	36%	37%	34%		41%	34%	36%	22%		41%	40%	34%	23%	20%		37%	35%	
*Daily*	14%	17%	12%		12%	15%	14%	17%		21%	12%	9%	9%	7%		10%	15%	
**Arepas**				0.117					0.005						0.076			0.561
*Monthly*	3%	2%	4%		4%	2%	3%	5%		3%	3%	2%	3%	4%		4%	3%	
*Weekly*	33%	31%	34%		42%	32%	28%	32%		29%	33%	39%	34%	33%		29%	34%	
*Daily*	64%	67%	62%		54%	66%	68%	63%		68%	64%	58%	62%	63%		67%	64%	
**Coffee**				0.012					0.036						< 0.001			0.943
*Monthly*	19%	17%	20%		21%	21%	17%	21%		26%	17%	11%	11%	16%		19%	19%	
*Weekly*	13%	15%	11%		17%	13%	10%	9%		17%	12%	9%	9%	7%		13%	12%	
*Daily*	69%	68%	69%		62%	66%	73%	70%		57%	71%	79%	80%	69%		68%	69%	
**Cheese**				0.0115					0.003						0.251			0.592
*Monthly*	5%	5%	5%		3%	5%	6%	12%		4%	5%	7%	6%	66%		6%	5%	
*Weekly*	37%	40%	35%		36%	35%	39%	43%		36%	38%	39%	36%	41%		40%	37%	
*Daily*	57%	54%	60%		61%	60%	55%	45%		60%	58%	54%	58%	52%		54%	58%	
**Vegetables**				0.0379					< 0.001						0.014			0.226
*Monthly*	12%	14%	11%		7%	8%	17%	17%		16%	8%	11%	11%	8%		15%	11%	
*Weekly*	57%	58%	57%		57%	59%	56%	59%		56%	58%	59%	56%	61%		62%	56%	
*Daily*	31%	29%	32%		36%	33%	27%	23%		28%	33%	30%	33%	31%		22%	32%	
**Fruits**				0.102					0.134						0.322			0.058
*Monthly*	18%	19%	17%		16%	16%	20%	25%		18%	18%	18%	18%	18%		15%	19%	
*Weekly*	61%	62%	60%		60%	61%	61%	56%		63%	61%	60%	59%	55%		67%	59%	
*Daily*	19%	19%	23%		24%	23%	19%	18%		19%	21%	21%	23%	28%		18%	22%	

^*^All estimates are weighted for complex survey design

^1^ SES was calculated using a version of the Graffar Scale modified for Venezuela [19], which combines income, profession, educational level, and housing conditions into a composite score

^2^ For individual food groups, p-values were calculated using Pearson’s chi-square, weighted for complex survey design. For DDS, p-values were calculated using Somers’ D, weighted for complex survey design

^3^ n = unweighted sample size

^4 ^DDS was calculated as an amended minimum dietary diversity-women (MDD-W) score, where each food category was given a score of 0 if consumed weekly or less or 1 if at least one portion was consumed daily. A final score was created by summing all eight categories. Food groups included in each category are listed in Appendix 1 in Table 4. Jackknife SE is reported adjusted for the primary sampling unit, parish

Food groups included in each category are listed in Additional file 2. Each food category was given a score of 0 if one to three portions were consumed weekly or less or 1 if one portion was consumed daily, and then a final score was created by summing all eight categories

Acronyms: SES: Socioeconomic status, DDS: Dietary Diversity Score, SE: Standard error

**Table 3:** Percent of Venezuelan adults consuming food groups by cardiometabolic risk status*

	Total	BMI Category^**1**^					Type 2 Diabetes^**2**^			Hypertension^**3**^		
		Under-weight	Normal weight	Overweight	Obesity	P-value^**4**^	No	Yes	P-value^**4**^	No	Yes	P-value^**4**^
**n**^**5**^	3402	133	1198	1204	853		2790	601		1908	1493	
**DDS**^**5**^						0.315			0.630			0.879
Mean (SE)	2.28 (0.07)	2.62 (0.17)	2.31 (0.08)	2.18 (0.08)	2.29 (0.08)		2.29 (0.06)	2.21 (0.14)		2.27 (0.07)	2.29 (0.08)	
**White Bread**						0.405			0.456			0.605
*Monthly*	10%	8%	10%	10%	9%		10%	10%		10%	10%	
*Weekly*	58%	57%	58%	61%	57%		59%	56%		59%	57%	
*Daily*	32%	36%	33%	29%	35%		32%	35%		31%	34%	
**Red Meat**						0.033			0.006			0.002
*Monthly*	19%	32%	18%	19%	17%		18%	24%		17%	22%	
*Weekly*	74%	58%	76%	75%	75%		75%	72%		76%	70%	
*Daily*	7%	10%	7%	6%	8%		7%	4%		7%	7%	
**Cookies**						0.481			0.848			0.214
*Monthly*	28%	18%	28%	28%	30%		28%	29%		27%	30%	
*Weekly*	53%	57%	54%	54%	50%		53%	52%		53%	53%	
*Daily*	19%	25%	19%	18%	20%		19%	18%		20%	17%	
**Soft drinks**						0.2916			< 0.001			< 0.001
*Monthly*	50%	51%	53%	50%	46%		48%	64%		46%	59%	
*Weekly*	36%	29%	35%	37%	35%		38%	24%		38%	29%	
*Daily*	14%	19%	13%	14%	14%		15%	13%		16%	11%	
**Arepas**						0.013			0.025			0.043
*Monthly*	3%	1%	4%	2%	3%		3%	2%		3%	3%	
*Weekly*	33%	25%	30%	33%	38%		32%	39%		31%	36%	
*Daily*	64%	74%	66%	65%	59%		65%	60%		66%	61%	
**Coffee**						0.789			0.009			0.075
*Monthly*	19%	18%	20%	17%	19%		20%	14%		20%	17%	
*Weekly*	13%	15%	13%	12%	12%		13%	8%		14%	10%	
*Daily*	69%	67%	67%	70%	69%		67%	78%		67%	73%	
**Cheese**						0.451			0.0929			0.875
*Monthly*	5%	5%	6%	5%	4%		5%	4%		14%	11%	
*Weekly*	37%	37%	40%	37%	35%		37%	42%		58%	57%	
*Daily*	57%	58%	55%	58%	61%		58%	54%		29%	32%	
**Vegetables**						0.011			0.478			0.5011
*Monthly*	12%	19%	14%	12%	7%		12%	11%		13%	11%	
*Weekly*	58%	59%	55%	58%	60%		58%	56%		57%	58%	
*Daily*	30%	22%	31%	30%	33%		30%	33%		30%	31%	
**Fruits**						0.099			0.8128			0.4633
*Monthly*	18%	15%	19%	17%	19%		18%	17%		17%	20%	
*Weekly*	61%	54%	58%	64%	61%		60%	62%		61%	59%	
*Daily*	21%	31%	24%	18%	20%		21%	21%		22%	21%	

^*^All estimates are weighted for complex survey design

^1^ BMI category was defined as weight (measured in kilograms) divided by height (measured in meters) squared, and classified as underweight (< 18.5 kg/m^2^), normal weight (18.5 to < 25.0 kg/m^2^), overweight (25.0 to < 30.0 kg/m^2^), or obesity (≥30.0 kg/m^2^) [21]

^2^Type 2 diabetes was defined by either: fasting plasma glucose was ≥126 mg/dL, 2-h after 75 g oral glucose tolerance test ≥200 mg/dL, or self-report of diabetes [24]

^3^Hypertension was defined as having a systolic blood pressure ≥ 140 mmHg, diastolic blood pressure ≥ 90 mmHg, or self-report of antihypertensive medication use [23]

^4^For individual food groups, p-values were calculated using Pearson’s chi-square, weighted for complex survey design. For DDS, *p*-values were calculated using Somers’ D, weighted for complex survey design

^5^ n = unweighted sample size

^6^DDS was calculated as an amended minimum dietary diversity-women (MDD-W) score, where each food category was given a score of 0 if consumed weekly or less or 1 if at least one portion was consumed daily. A final score was created by summing all eight categories. Food groups included in each category are listed in Appendix 1 in Table 4. Jackknife SE is reported adjusted for the primary sampling unit, parish

Acronyms: BMI: body mass index, DDS: Dietary Diversity Score, SE: Standard error

Daily consumption patterns for a number of food groups differed by sex, namely white bread, cookies, soft drinks, cheese, and vegetables. Compared to females, a higher proportion of males reporting daily consumption of white bread (35% of males vs 29% of females; *p* = 0.005) and soft drinks (17% vs 12% *p* = 0.002). However, females consumed more cookies (18% of males vs 20% of females; *p* = 0.0154), cheese (54% vs 60%; *p* = 0.0115), and vegetables (29% vs 32%; *p* = 0.0379) daily. There was no difference by sex for consumption patterns of fruit (*p* = 0.102) or arepas (*p* = 0.117).

Consumption patterns of white bread (*p* = 0.126), cookies (*p* = 0.963), soft drinks (*p* = 0.081), and fruits (0.134) did not vary by SES. Participants with higher SES consumed more red meat weekly compared to those with lower SES (*p* < 0.001): weekly consumption was 80% among those in the high & medium-high and medium categories, 69% among those in relative poverty, and 60% among those in extreme poverty. A similar pattern was observed for daily consumption of cheese (*p* = 0.003) and vegetables (*p* < 0.0001) where daily consumption was highest among those with high & medium-high SES (61% for cheese and 36% for vegetables in the highest SES category) compared to consumption among those in lower SES categories (45% for cheese and 23% for vegetables in the lowest SES category). However, consumption patterns were less linear for daily consumption of coffee and arepas. Daily arepa consumption was highest among participants in relative poverty (68%) and medium SES (66%), followed by participants in extreme poverty (63%), compared to participants in the high & medium-high category (54%) (*p* = 0.005). Generally, lower SES category correlated with higher daily intake of coffee (*p* = 0.036): daily coffee consumption was 73% among those in relative poverty and 70% among those in extreme poverty, compared to 66% among those in the medium SES category and 62% in the high and medium-high category.

Consumption patterns of white bread (*p* = 0.293), cheese (*p* = 0.251), and fruits (*p* = 0.322) did not vary by age category. However, certain food groups were consumed more frequently by younger age groups compared to others. For instance, younger participants consumed more red meat weekly compared to older participants (*p* < 0.001): weekly consumption was 78% among those < 35-years-old and 75% among 45–54-year-olds, compared to 61% among > 65-year-olds. Similarly, younger participants consumed more soft drinks weekly than older participants (p < 0.001): weekly consumption was 41% among those < 35-years-old, compared to 23% among 55–64-year-olds and 20% among > 65-year-olds. Weekly vegetable consumption, however, was higher among older age categories (*p* = 0.014) with 61% of those > 65 years, 56% of those 55–65 years, and 59% of those 45–54 years consuming vegetables weekly, compared to 58% of those 35–44 and 56% < 35 years of age.

The majority of food group consumption did not vary significantly by locality, with the exception of red meat (*p* = 0.003). Red meat was most likely to be consumed weekly among those living in urban areas (76%) compared to those in rural areas (67%). Fruit consumption bordered statistical significance (*p* = 0.058) and at least one portion of fruit was more likely to be consumed weekly in rural areas (67%) compared to urban areas (59%).

### Distribution of consumption by cardiometabolic risk status

DDS did not differ by cardiometabolic risk status. Frequency of food group intake did differ by BMI category for red meat (*p* = 0.033), arepas (*p* = 0.013), and vegetables (*p* = 0.011). Daily consumption of red meat and arepas generally decreased with increasing BMI category. For red meat, 10% of participants with an underweight BMI reported daily consumption and 7% of participants with normal weight, compared to 6% of participants with an overweight BMI. Participants with obesity, however, had slightly higher red meat consumption with 8% of participants reporting daily consumption. Daily arepa consumption decreased with increasing BMI category, with 74% of participants with an underweight BMI consuming arepas daily, compared to 66% with a normal BMI, 65% with an overweight BMI, and 59% with obesity. One-third of participants with obesity had daily vegetable intake compared to 30% with an overweight BMI and 22% of participants with an underweight BMI. There was no difference in vegetable intake by hypertension (*p* = 0.57) or T2D status (*p* = 0.68).

Consumption of red meat, soft drinks, arepas, and coffee differed significantly by T2D status. Those with T2D were less likely to consume red meat, soft drinks, and arepas daily: 4% of participants with T2D consumed red meat weekly compared to 7% without T2D (*p* = 0.006), 13% of those with T2D consumed soft drinks daily compared to 15% without (*p* < 0.001), and 60% of those with T2D consumed arepas daily compared 65% of those without. Daily coffee consumption, however, was more frequent among participants with T2D (78%) compared to those without T2D (67%).

Similarly, consumption of red meat, soft drinks, and arepas differed significantly by hypertension status. While daily consumption of red meat was 7% for both those participants with hypertension and those without, weekly consumption was 76% among those without hypertension compared to 70% among those with hypertension (*p* = 0.002). Daily consumption of soft drinks (p < 0.001) and arepas (0.043) were both higher among participants without hypertension: 16% of those without hypertension consumed soft drinks daily compared to 11% of those with hypertension, and 66% of those without hypertension consumed arepas daily compared to 61% of those with hypertension.

## Discussion

This nationally representative descriptive analysis of dietary patterns of Venezuelans in 2014–2017 found that the study population had a high prevalence of obesity, T2D, and hypertension. Most frequently consumed food groups included white bread, arepas, coffee, and cheese. Intake of many Western foods were relatively low, with over 75% of participants consuming french fries, burgers, and fast foods only monthly or less frequently.

In general, this analysis found females had healthier diets than males, with lower consumption of white bread, red meat, and soft drinks, although dietary diversity was similar between the sexes. There were inconsistent differences in intake by SES category: those with higher SES, compared to those in relative or extreme poverty, consumed more portions of some healthy foods (e.g. higher daily intake of fruit and vegetables), but also unhealthy foods (e.g. higher daily intake of white bread and cheese). Overall, younger and urban Venezuelans ate more Western foods than both older and rural Venezuelans.

Overall, dietary diversity was very low. Gomez et al. (2019), a study of eight Latin American countries, found that other countries have much more diverse diets with DDS scores of five to six (out of nine) [15]. They found a slightly higher mean score in Venezuela than this analysis (5.62 of 9 compared to 2.3 of 8), but their study was conducted in only urban areas and during an earlier time period (2014–2015) than EVESCAM (2014–2017) which may have been affected by the onset of the humanitarian crisis in Venezuela.

Healthier food groups did not differ substantially by BMI category or T2D or hypertension status, while unhealthier foods (namely red meat, cheese, and arepas) were consumed more frequently by participants classified with overweight BMI and obesity, but less frequently by individuals with T2D and hypertension. This may suggest that people with diagnosed T2D and hypertension may follow positive behaviour change and nutritional recommendations made upon diagnosis [26]. Furthermore, individuals with obesity consumed more vegetables daily and consumed fewer soft drinks and cookies, potentially reflecting attempts to lose weight.

Although consumption of Western foods was low, the most commonly consumed food groups are not considered ‘healthy’ by most dietary guidelines for the region and worldwide [27]. White bread, arepas, and cheese dominated daily consumption in this nationally representative sample. In general, the composition of the cheeses in Venezuela have high fat content [13] and may be a factor contributing to obesity and T2D. In fact, a recent study in the US found that increasing cheese consumption by > 0.5 servings per day was associated with 9% (95% CI: 2–16) higher risk of T2D, compared to maintaining usual cheese consumption [28].

These results suggest that the nutrition transition has not influenced the dietary intake of Venezuela as markedly as other Latin American countries, such as Brazil and Mexico [5, 8]. This could be due to the economic crisis in Venezuela, which started in 2014 and led to government restrictions on foreign products [29]. Meanwhile, traditional foods have long been subsidized by the government, possibly increasing their availability and consumption. Arepas, in particular, are considered a staple food in Venezuela. They are prepared from a cornmeal that is fortified with vitamin A and iron. The glycemic index is relatively high (74), though lower than white wheat bread (98) [30], however, to our knowledge, no studies have specifically evaluated the prospective association between arepas intake and obesity or T2D. However, given strong evidence linking refined grains to these outcomes [31], one might posit that arepas may also increase risk and substituting for whole grains may decrease risk. Unfortunately, it is unlikely that such substitutions would be culturally acceptable.

Despite markedly low consumption of Western, ultra-processed foods, the prevalence of obesity (24.6%) was higher in this sample than the regional average: in 2014, 19% of adults in Latin America were classified with obesity [32]. However, adult obesity prevalence remains lower in Venezuela than in Mexico or Chile, both with an obesity prevalence of approximately 35% in 2014 [33]. Furthermore, obesity prevalence found in this sample is lower than previous estimates from before the socio-political crisis. For example, the weighted prevalence of obesity was 27.9%, when calculated from all population-based studies published between 1999 and 2010 which included a total of 8584 individuals [13]. In a previous regional analysis that included the EVESCAM cohort but did not include any dietary data, obesity prevalence in Venezuela did not vary by urban versus rural areas and was generally highest in middle quartiles of educational attainment and lowest in the bottom quartile [7].

In the present study, obesity prevalence was higher among women than men (26.7% versus 22.2%), a trend that matches obesity patterns worldwide and in other parts of Latin America [5, 7]. Although some aspects of the diet in this Venezuelan cohort were healthier among women, the higher prevalence of obesity could be explained by several factors including body size preferences for women in Latino cultures [34], lower physical activity levels (particularly work-related physical activity) [35], parity and resultant excess gestational weight gain/post-partum weight retention, and potentially genetic and/or hormonal differences [7].

While prevalence for hypertension in our study was comparable to previous studies, the estimates for diabetes were higher. Here, the weighted prevalence was 13.1% (95% CI: 11.2–15.7), for T2D and 30.8% (27.7–34.0) for hypertension. Other studies reported diabetes prevalence to be about 8% and hypertension to be about 30% [36, 37]. Differences in definitions of T2D and sampling may explain some of these differences, however this warrants further study using longitudinal data.

This study has a number of strengths. First, EVESCAM is the first nationally representative study of Venezuelan diet and provides a better understanding of the nuances in dietary patterns throughout Latin America, particularly in a country undergoing a socio-political crisis and that has been neglected in global nutrition literature. This analysis provides nationally-representative estimates of diabetes, obesity, and hypertension prevalence during a time period where health data has been sparse in Venezuela. Further, a number of diverse traditional food groups specific to the study context, such as arepas, empanadas, and fried bananas, were included in the questionnaire, as well as pictures of portion sizes to ensure that participants could more accurately self-report the frequency and number of portions consumed.

Nonetheless, study design limitations must be taken into consideration. First, we cannot definitively rule out type 1 diabetes cases but globally over 95% of diabetes cases are T2D [38]. In this sample, however, very few participants were on insulin (7%) and average age of diagnosis was 50.6 years, so this was unlikely to be a major source of error in this analysis. Second, this cross-sectional study relied on self-reporting of semi-quantitative nutritional data, which is prone to recall bias and impedes the ability to make causal inferences from this analysis. The semi-quantitative nature of the questionnaire also limited our ability to calculate caloric intake from each food group. In general, countries around the world are increasingly shifting towards food-based dietary guidelines [39]. It was outside the aims and scope of this study to explore nutrient data such as energy and so the instrument used was not suitable to capture nutrient data. Finally, the questionnaire used organized answer options in a manner that may have increased measurement error. Specifically, frequency of consumption was in increasing order within daily, weekly, and monthly categories. However, this questionnaire was completed with the guidance of strictly trained enumerators who supervised that answer choices matched the participants’ consumption patterns using showcards with portion sizes.

Further studies that employ validated quantitative methods to measure diet may assist in assessing diet more accurately, as well as conducting nutrient analysis. Moreover, future studies could also include recipes and preparation styles to better disaggregate ingredients included in mixed dishes. Lastly, since this study is conducted in a vulnerable population undergoing a humanitarian crisis, longitudinal data is needed to understand how food patterns have changed since baseline data collection in 2014–2017, when the crisis was already ongoing but less extensive. Diets might have changed among individuals with new cases of T2D or hypertension or with exacerbated complications of these conditions. Furthermore, though obesity was clearly a national problem in Venezuela at the time of data collection, this may have changed with food insecurity.

## Conclusions

In summary, this nationally representative, cross-sectional analysis suggests that that dietary intake Venezuela are quite different from other countries in Latin America which have a high reliance on soft drinks and ultra-processed foods. Nutrition policy measures throughout Latin America should be tailored to consumption patterns and socio-political contexts of each country.

### Supplementary information

**Additional file 1.** Dietary Intake Questionnaire.

**Additional file 2.** Show Cards Used in the EVESCAM Study.

## Data Availability

The datasets used and/or analysed during the current study are available from the corresponding author on reasonable request.

## Appendix 1

**Table 4:** Food Categories Used for Dietary Diversity Score

Food Category for MDD-W	Food Group in EVESCAM DDS
Grains, white roots and tubes, plantains	White bread, pasta, cereal, oats, rice, potato, plantain, yucca
Pulses	Legumes
Nuts & Seeds	Nuts
Dairy products	Milk, cheese
Animal-based foods	Fish, poultry, red meat, hamburgers
Eggs	Eggs
Other Fruits	Fruit
Vitamin-A rich fruits	
Other Vegetables	Vegetables
Leafy green vegetables	

## Abbreviations

BMI: Body mass index
CI: Confidence Interval
DALYs: Disability-adjusted life years
DDS: Dietary Diversity Score
EVESCAM: Estudio Venezolano de Salud Cardio-Metabólica
IQR: Interquartile ranges
MDD-W: Minimum Dietary Diversity-Women
NCDs: Non-communicable diseases
OGTT: Oral glucose tolerance test
SES: Socioeconomic status
