# Supplementary material for: Dietary intake and cardiometabolic risk factors among Venezuelan adults: a nationally representative analysis
Source: BMC Nutr. 2020 Oct 16;6:61. doi: 10.1186/s40795-020-00362-7 (PMC7566137; doi:10.1186/s40795-020-00362-7)
Supplement: Supplementary file 1 — Additional file 1. Dietary Intake Questionnaire. [file 40795_2020_362_MOESM1_ESM.docx]

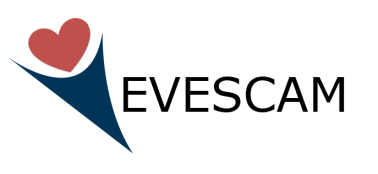
Appendix 1: Dietary Intake Questionnaire

**Sociedad Venezolana de Medicina Interna**

**Estudio Venezolano de Salud Cardiometabólica**

**Fundación para la Investigación Clínica, salud Pública y Epidemiológica de Venezuela (FISPEVen)**

**QUESTIONNAIRE 6.  FOOD-FREQUENCY QUESTIONNAIRE**

| Label | Please check the food list below. If you eat these foods, compare the amount you eat with the serving size shown in the photo, and report how many servings you typically consume in a day, week or month according to the case. You will be guided by a trained staff. |  |  |  |  |  |  |  |  |
| --- | --- | --- | --- | --- | --- | --- | --- | --- | --- |
| **FOOD GROUP** | **PORTION SIZE (photo)** | **NUMBER OF PORTIONS** | | | | | | | |
|  |  | **Daily** | | | **Weekly** | | | **Last month** | |
|  |  | **one** | **2 to 4** | **5 or**  **more** | **one** | **2 to 4** | **5 or**  **more** | **0** | **1 to 3** |
| **Fruits**  1. Whole or chopped fruit | 1 medium unit  1 large cup (240 cc) of chopped fruit |  |  |  |  |  |  |  |  |
| 2. Fruit juice | 1 glass of fruit juice (200 cc) |  |  |  |  |  |  |  |  |
| 3. **Vegetables**(ask salads) | - Raw: 1 large cup (8 ounces = 240 cc)  - Cooked: ½ large cup |  |  |  |  |  |  |  |  |
| **Dairy products and derivatives**:  4. Milk and whey | - Milk: 1 glass (200 cc)  - Whey: 1 small glass (180 cc) |  |  |  |  |  |  |  |  |
| 5. Cheeses | - 1 slice (30 g)  - 3 tablespoons (30 g, that is 10 g per unit) |  |  |  |  |  |  |  |  |
| 6. **Fats**  (butter, margarine, milk cream, oil) | - Butter, margarine, mayonnaise and cream of milk: 1 tablespoon (15 g)  - Oils: 1 tablespoon (10 cc = 10 g) |  |  |  |  |  |  |  |  |
| **Starches**  7. White bread | 1 slice of bread = ½ french bread |  |  |  |  |  |  |  |  |
| 8. Arepa | 1 medium unit (100 g) |  |  |  |  |  |  |  |  |
| 9. Pasta | 1 cup or 1 small plate (150 g) |  |  |  |  |  |  |  |  |
| 10. Cooked white rice | 1 cup (125 g) |  |  |  |  |  |  |  |  |
| 12. Cereals | ½ cup of flakes |  |  |  |  |  |  |  |  |

| 13. Boiled yuca and / or casava | - Yucca: 1 medium piece (150 g)  - Casabe: ¼ medium cake |  |  |  | | |  |  |  |  |  |
| --- | --- | --- | --- | --- | --- | --- | --- | --- | --- | --- | --- |
| 14. Whole potato or mash      ed | 1 small potato (50 g) = ½ cup of mash |  |  |  | | |  |  |  |  |  |
| 15. Plantain | Medium plantain |  |  |  | | |  |  |  |  |  |
| **Whole grains**  16. Prepared flaked oatmeal | ½ cup cooked |  |  |  | | |  |  |  |  |  |
| 17. Stacked corn cachapa | ½  small cachapa (80 g) |  |  |  | | |  |  |  |  |  |
| 18. **Legumes**(beans, lentils, vetch, etc.) | 1 cup cooked (210 g) |  |  |  | | |  |  |  |  |  |
| 19. **Soups**(not including legumes) | 1 cup (240 g) |  |  | |  |  | |  |  |  |  |
| 20. **Nuts and seeds**(nuts, peanuts, pistachios, hazelnuts, almonds) | ½ cup or 3 tablespoons (25 g) |  |  | |  |  | |  |  |  |  |
| 21. **Fish**(include sardine and canned tuna) | - Fish: 1 medium steak (120 g)  - Sardine and tuna: 1 drained can (120 g) |  |  | |  |  | |  |  |  |  |
| 22. **Poultry**(Chicken, chicken) | - Chicken: 1 breast or 2 thighs (120 g) |  |  | |  |  | |  |  |  |  |
| 24. **Eggs** | 1 unit (50 g) |  |  | |  |  | |  |  |  |  |
| 25. **Meats** | 1 medium steak (120 g) |  |  | |  |  | |  |  |  |  |
| **Fried food**  26. Empanadas | 1 medium unit (80 g) |  |  | |  |  | |  |  |  |  |
| 27. French fries | ½ cup (68 g) |  |  | |  |  | |  |  |  |  |

| Fried banana slices or tostones | Slice: ½ banana = 4 units (150 g)  Tostones ½ banana = 4 units (150 g) |  |  |  |  |  |  |  |  |
| --- | --- | --- | --- | --- | --- | --- | --- | --- | --- |
| **28. Fast food**  Burgers  Hot dogs  Pepitos (tenderloin sandwich)  Pizzas | 1 small unit (102 gr) or 1 pc.  1 medium unit (medium Frankfurt sausage = 30 g)   1. ½ unidad de ½ pan canilla = ¼ pan canilla 2. ½ unit of ½ bread quill = ¼ bread quill   ½ small pizza |  |  |  |  |  |  |  |  |
| 29. Sweet or savory cookies | - Sweet cookie: 1 package (30 g)  - Soda cookie: 1 package |  |  |  |  |  |  |  |  |
| 30. Cakes or desserts | - Sweets and desserts: ½ cup = 1 ounce (33 g) |  |  |  |  |  |  |  |  |
| 31. Sugar | 1 tablespoon = 15 g  1 teaspoon = 5 g |  |  |  |  |  |  |  |  |
| 32. Meals in Fast Food Sales (include franchises and street shops) | Write down the number of times the subject eats in those establishments |  |  |  |  |  |  |  |  |
| 33. **Beverages**(Soft drinks and instant drinks like cold tea) | 1 glass = 240 cc = 8 ounces = 1 bottle |  |  |  |  |  |  |  |  |
| 34. **Water** | 1 glass = 240 cc = 8 ounces |  |  |  |  |  |  |  |  |
| 35. **Alcohol** | Beer (360cc = 1 and a half) Wine (150 cc = 1 glass) Whiskey, rum and others (1.5 ounces = 45 cc) |  |  |  |  |  |  |  |  |
| 36. **Coffee** | Coffee = 1 medium cup |  |  |  |  |  |  |  |  |
