# Supplementary material for: Dietary intake and cardiometabolic risk factors among Venezuelan adults: a nationally representative analysis
Source: BMC Nutr. 2020 Oct 16;6:61. doi: 10.1186/s40795-020-00362-7 (PMC7566137; doi:10.1186/s40795-020-00362-7)
Supplement: Supplementary file 2 — Additional file 2. Show Cards Used in the EVESCAM Study. [file 40795_2020_362_MOESM2_ESM.pdf]

Estimado Participante del estudio **EVESCAM**, a continuación le vamos a preguntar acerca de sus hábitos de alimentación. Para esto, deseamos conocer la **cantidad de raciones** que consume de los diferentes alimentos aquí presentados.

Las imágenes mostradas a continuación representan el tamaño de **1 ración** del alimento. Por lo que debe contestar cuántas de estas raciones consume diariamente, semanalmente o mensualmente.

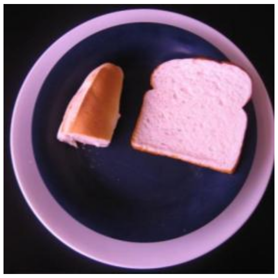

01 ¿Cuántas de estas raciones consume diariamente, semanalmente o mensualmente?

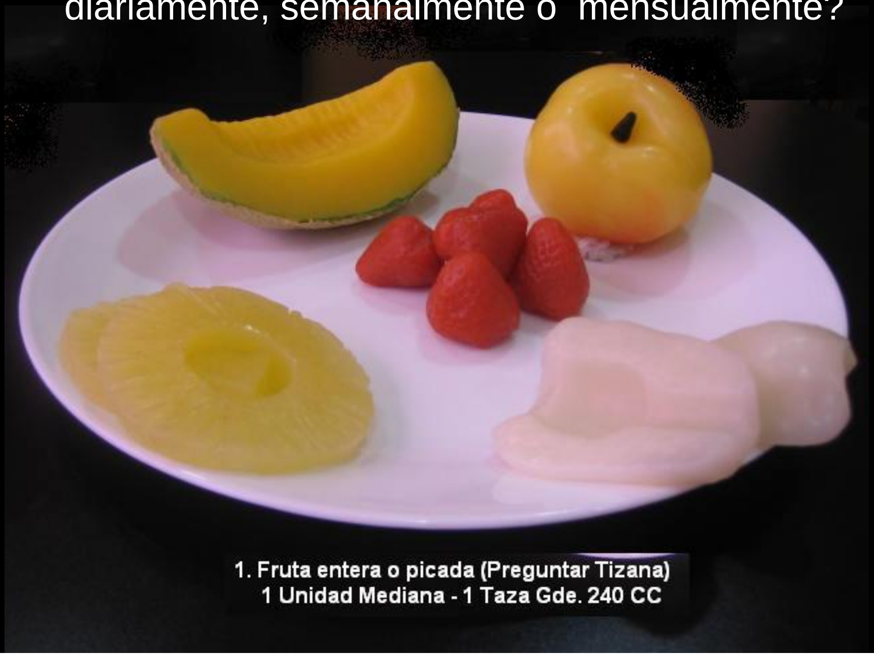

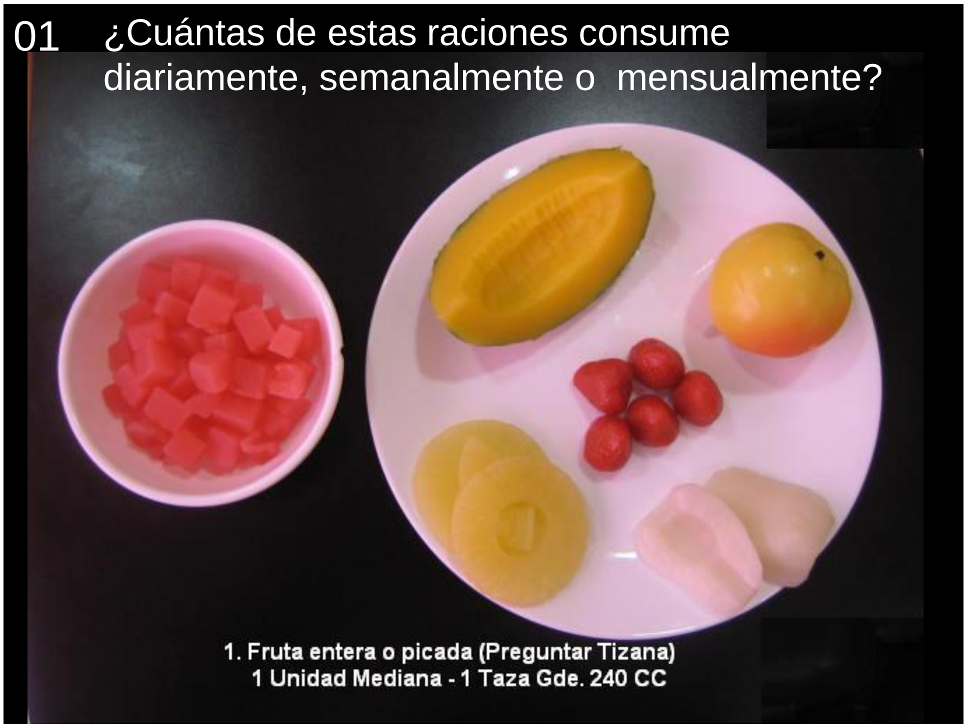
01
¿Cuántas de estas raciones consume diariamente, semanalmente o mensualmente?
1. Fruta entera o picada (Preguntar Tizana)
1 Unidad Mediana - 1 Taza Gde. 240 CC

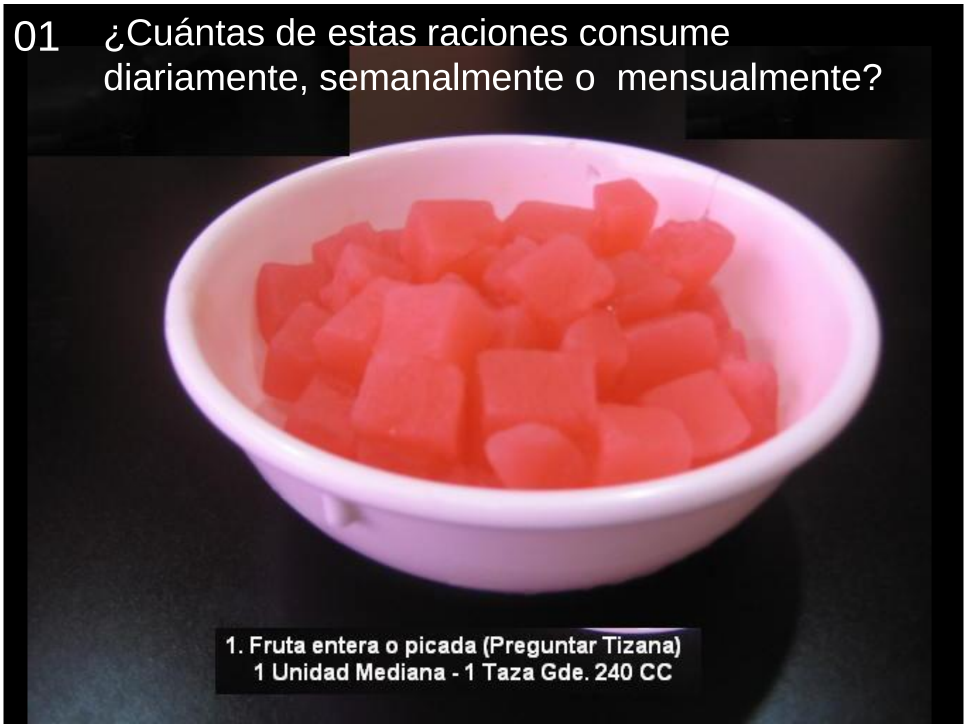
01
¿Cuántas de estas raciones consume diariamente, semanalmente o mensualmente?
1. Fruta entera o picada (Preguntar Tizana)
1 Unidad Mediana - 1 Taza Gde. 240 CC

02 ¿Cuántas de estas raciones consume diariamente, semanalmente o mensualmente?

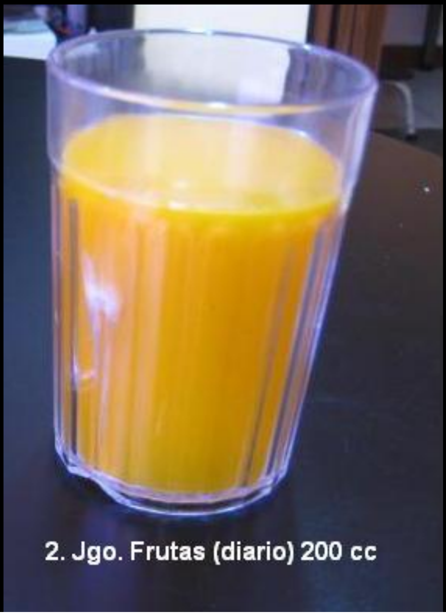

02 ¿Cuántas de estas raciones consume diariamente, semanalmente o mensualmente?

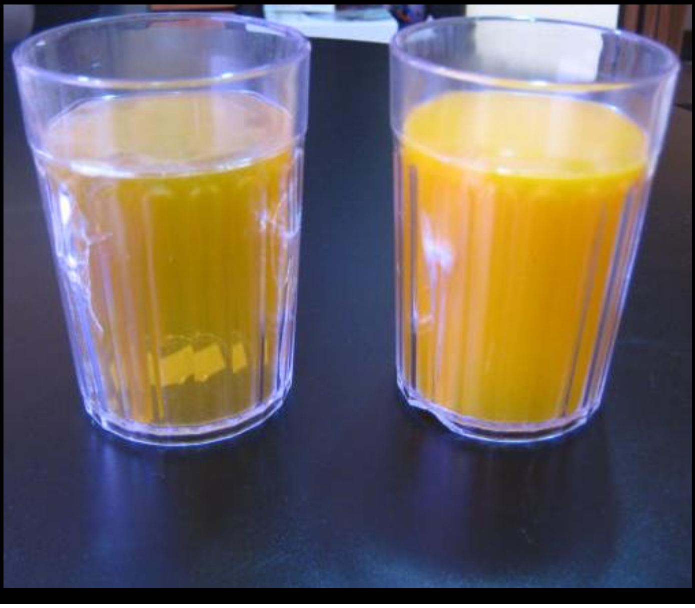

03 ¿Cuántas de estas raciones consume diariamente, semanalmente o mensualmente?

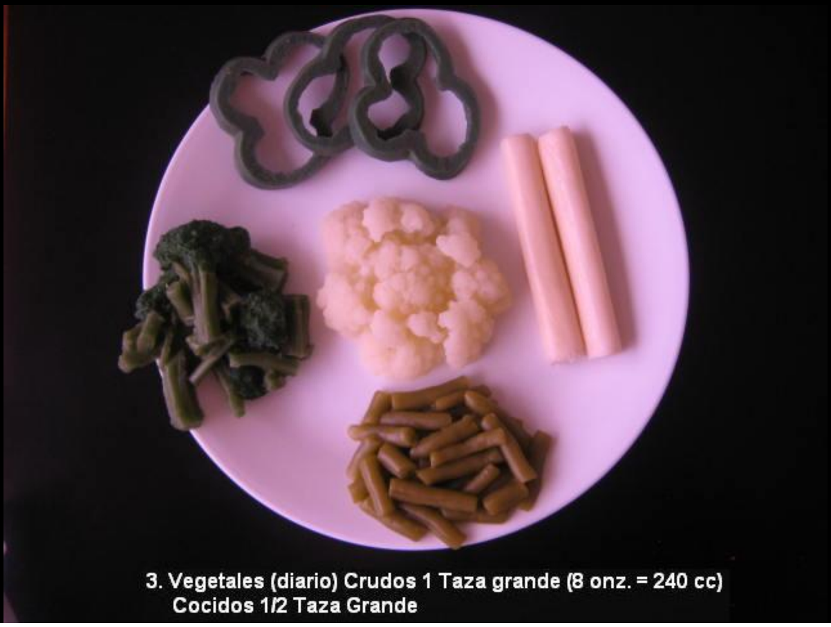

03 ¿Cuántas de estas raciones consume diariamente, semanalmente o mensualmente?

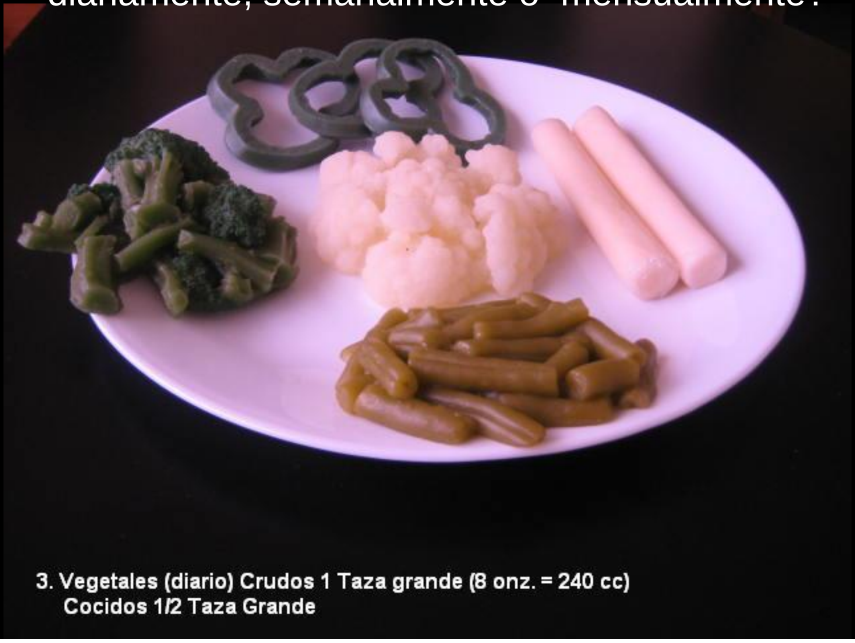

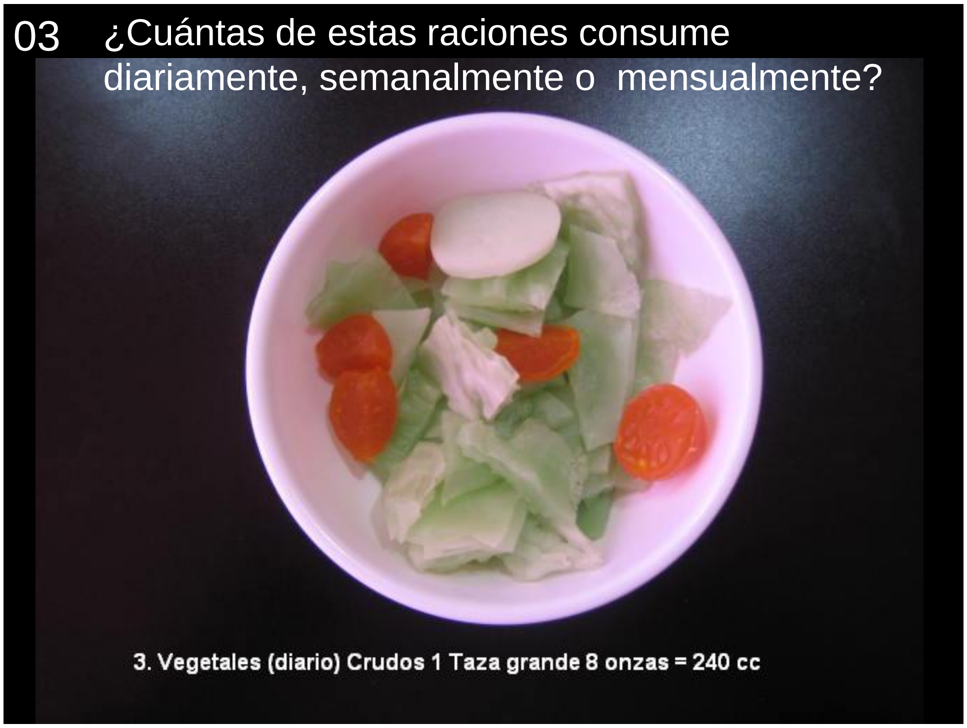
03
¿Cuántas de estas raciones consume diariamente, semanalmente o mensualmente?
3. Vegetales (diario) Crudos 1 Taza grande 8 onzas = 240 cc

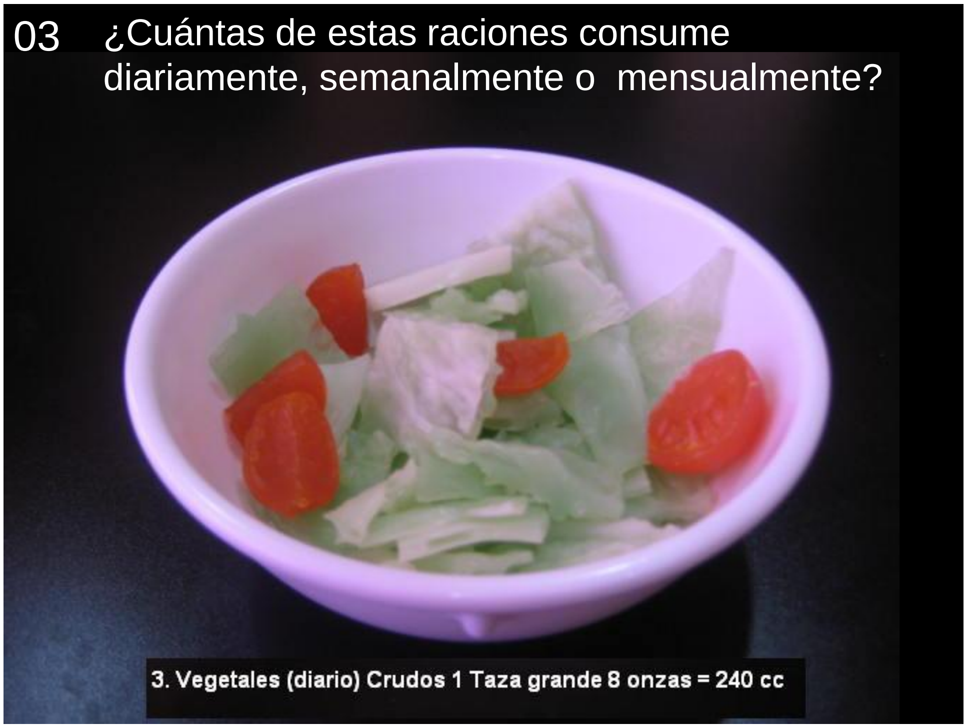
03
¿Cuántas de estas raciones consume diariamente, semanalmente o mensualmente?
3. Vegetales (diario) Crudos 1 Taza grande 8 onzas = 240 cc

04 ¿Cuántas de estas raciones consume diariamente, semanalmente o mensualmente?

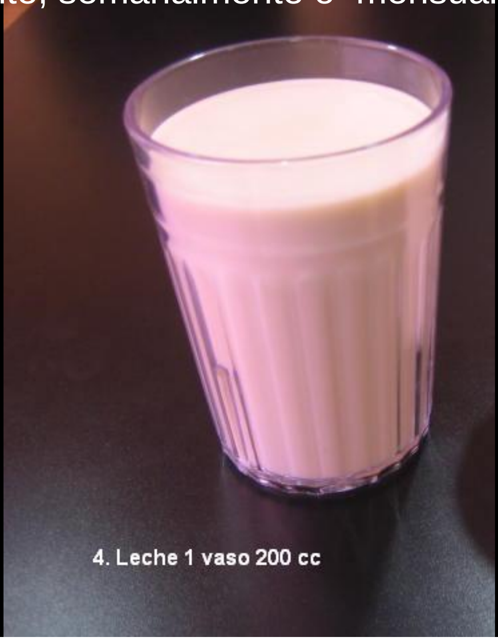

05 ¿Cuántas de estas raciones consume diariamente, semanalmente o mensualmente?

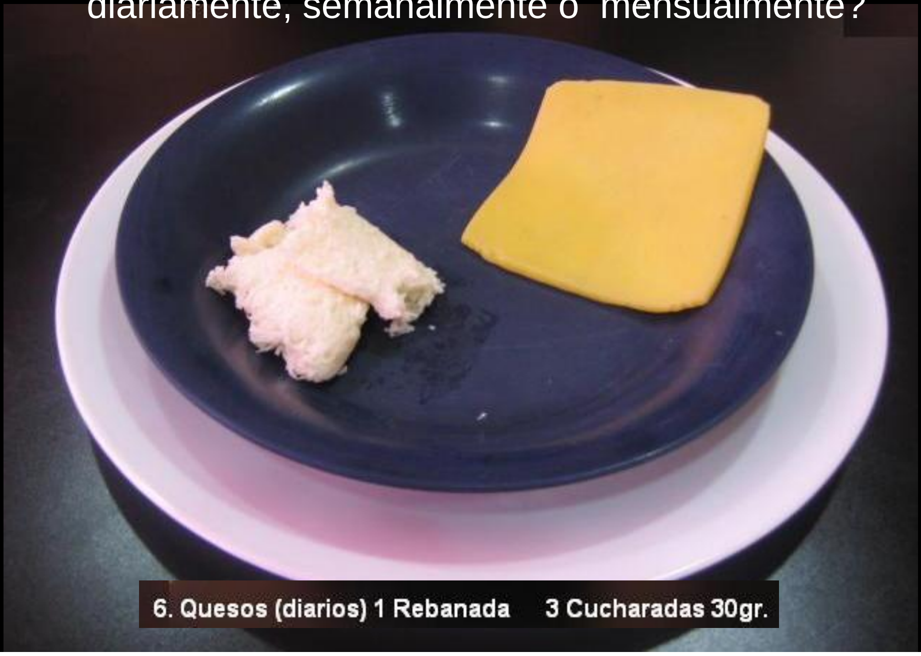

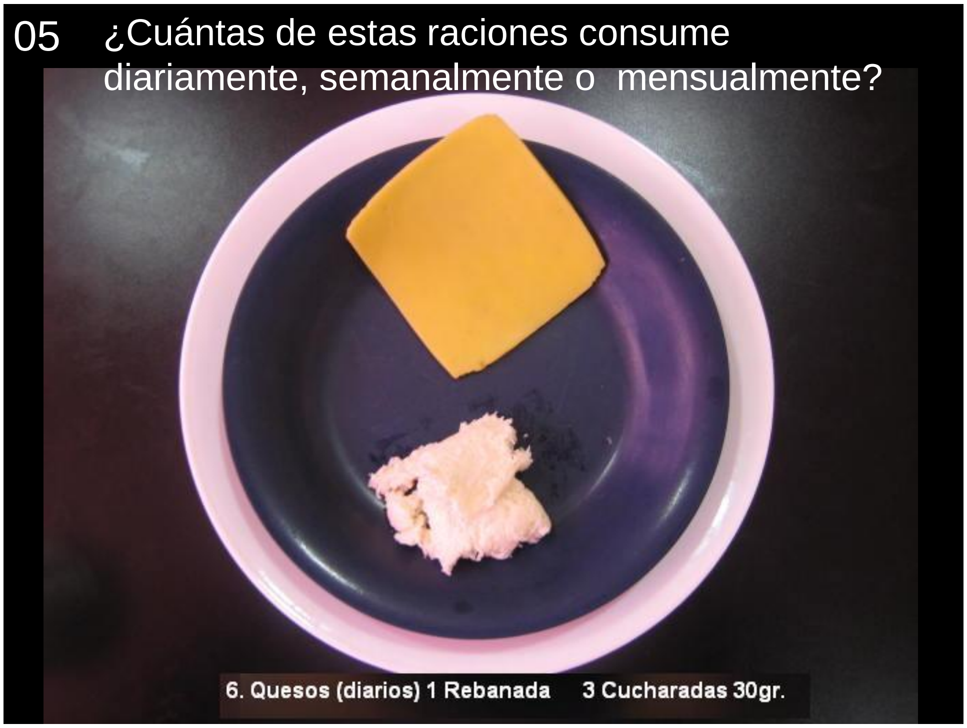
05
¿Cuántas de estas raciones consume diariamente, semanalmente o mensualmente?
6. Quesos (diarios) 1 Rebanada 3 Cucharadas 30gr.

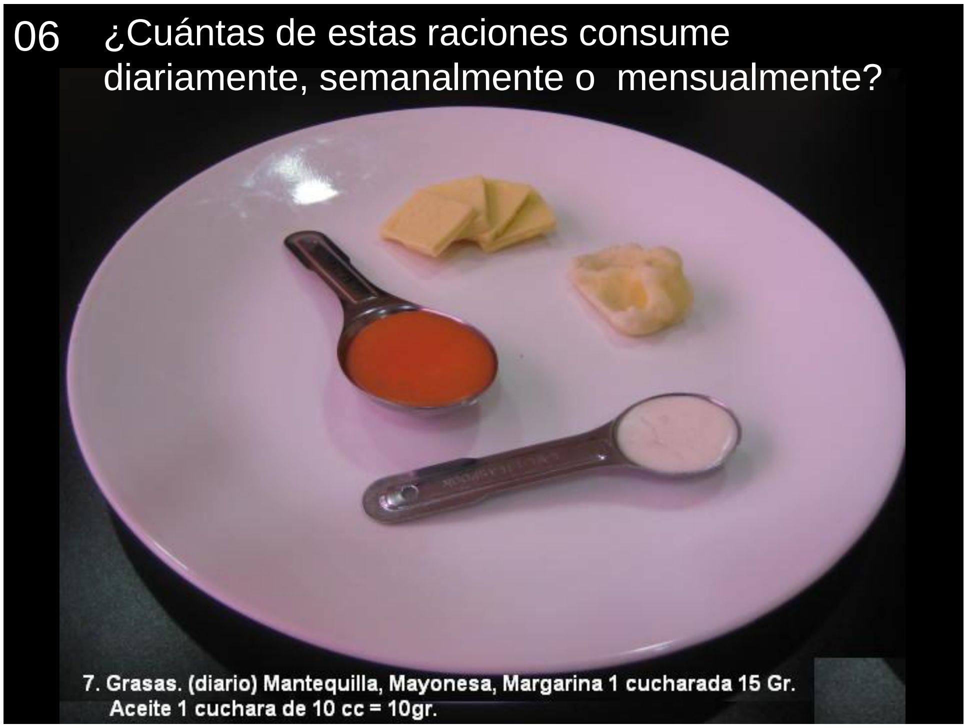
06
¿Cuántas de estas raciones consume diariamente, semanalmente o mensualmente?
7. Grasas. (diario) Mantequilla, Mayonesa, Margarina 1 cucharada 15 Gr.
Aceite 1 cuchara de 10 cc = 10gr.

06 ¿Cuántas de estas raciones consume diariamente, semanalmente o mensualmente?

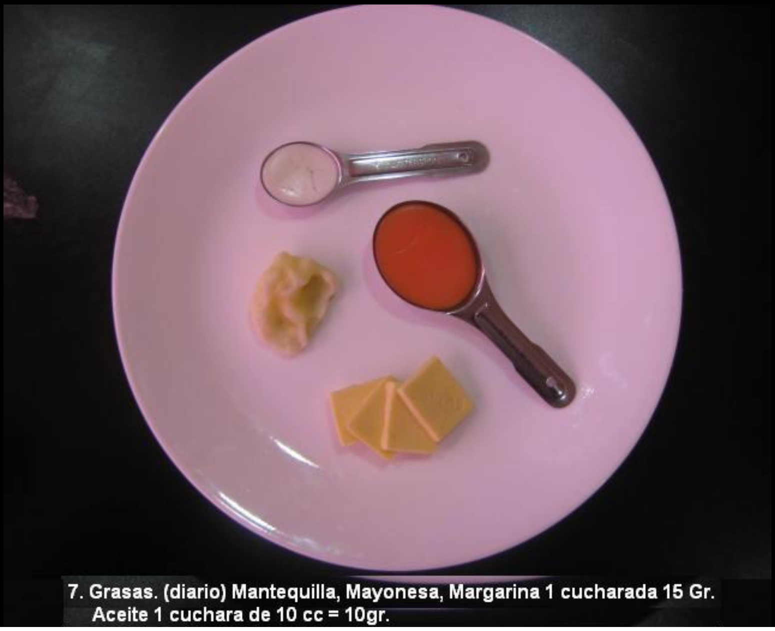

07 ¿Cuántas de estas raciones consume diariamente, semanalmente o mensualmente?

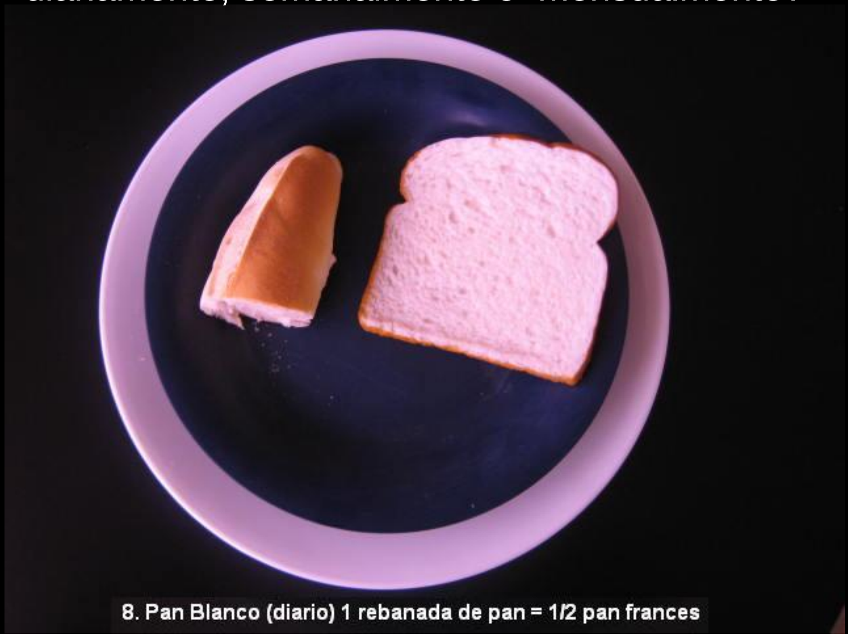

07 ¿Cuántas de estas raciones consume diariamente, semanalmente o mensualmente?

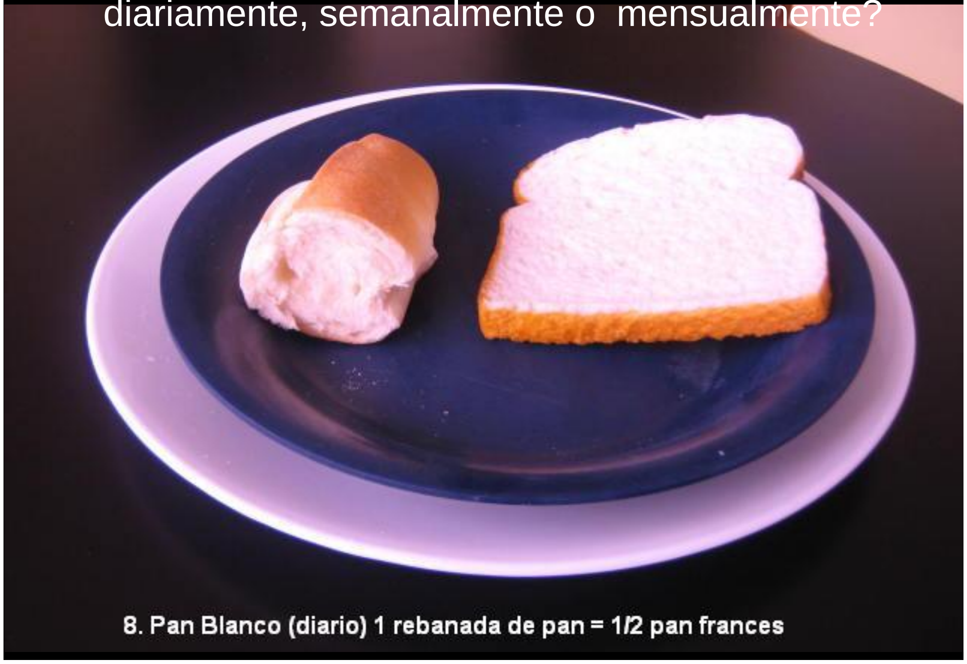

8. Pan Blanco (diario) 1 rebanada de pan = 1/2 pan frances

08 ¿Cuántas de estas raciones consume diariamente, semanalmente o mensualmente?

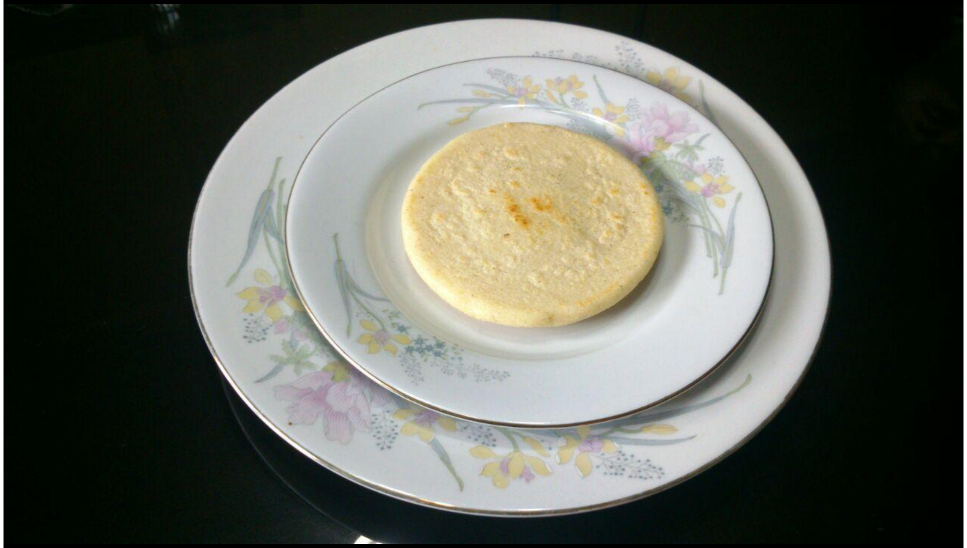

1 unidad mediana (100 g)

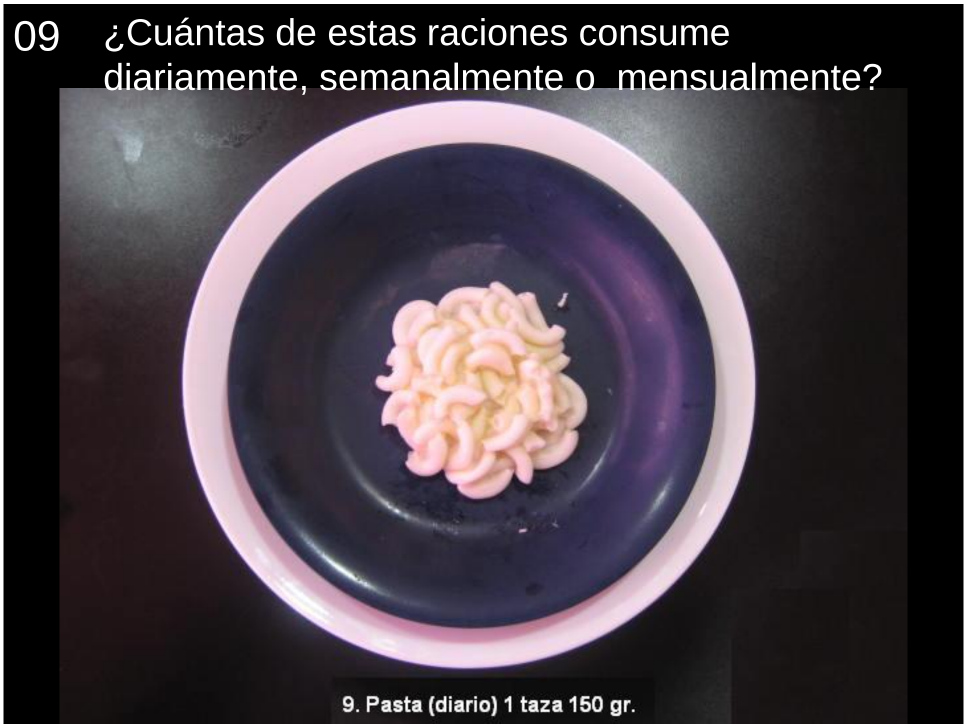
09
¿Cuántas de estas raciones consume diariamente, semanalmente o mensualmente?
9. Pasta (diario) 1 taza 150 gr.

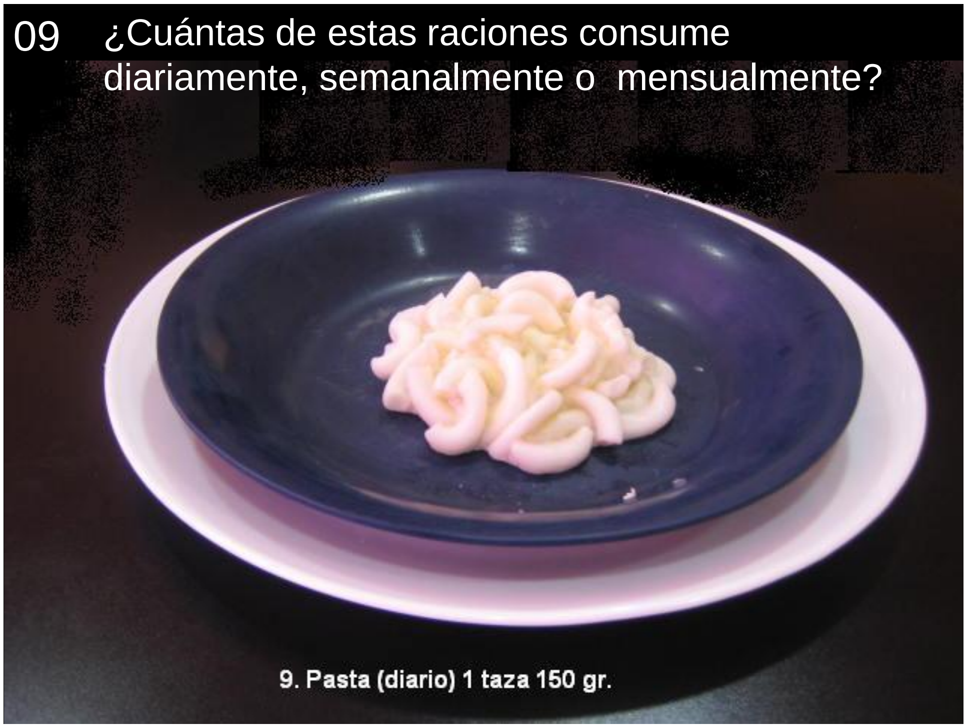
09
¿Cuántas de estas raciones consume diariamente, semanalmente o mensualmente?
9. Pasta (diario) 1 taza 150 gr.

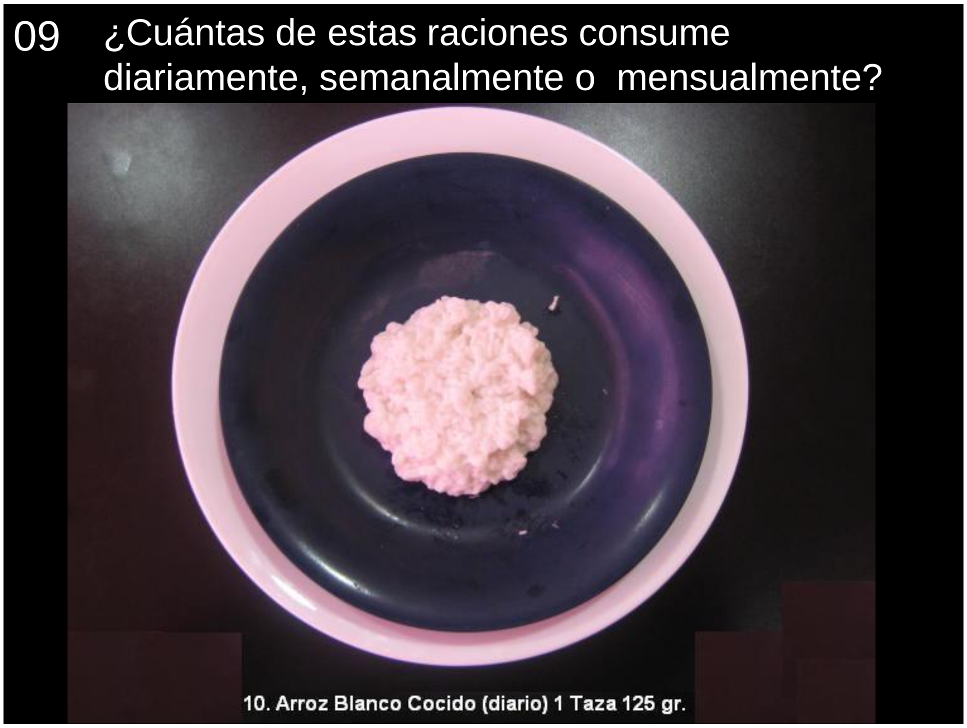
09
¿Cuántas de estas raciones consume diariamente, semanalmente o mensualmente?
10. Arroz Blanco Cocido (diario) 1 Taza 125 gr.

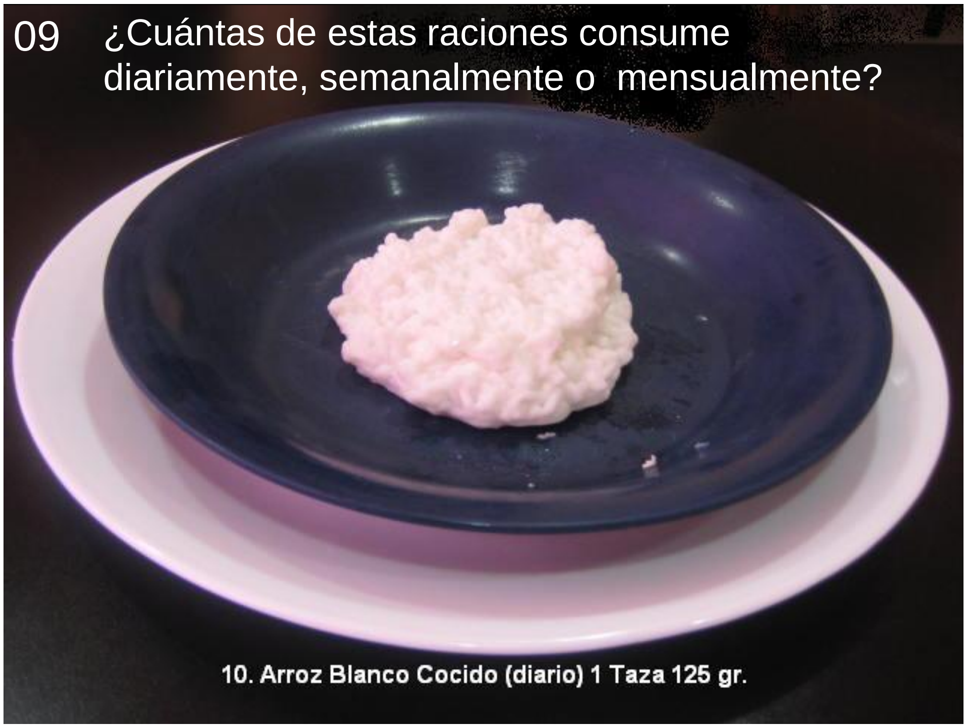
09
¿Cuántas de estas raciones consume diariamente, semanalmente o mensualmente?
10. Arroz Blanco Cocido (diario) 1 Taza 125 gr.

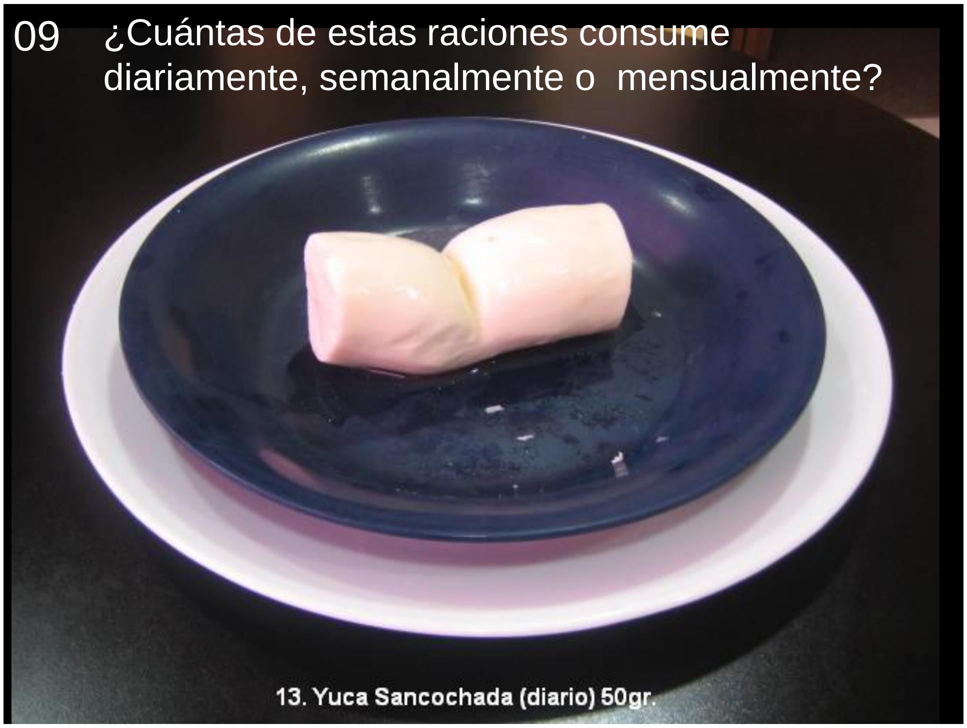
09
¿Cuántas de estas raciones consume diariamente, semanalmente o mensualmente?
13. Yuca Sancochada (diario) 50gr.

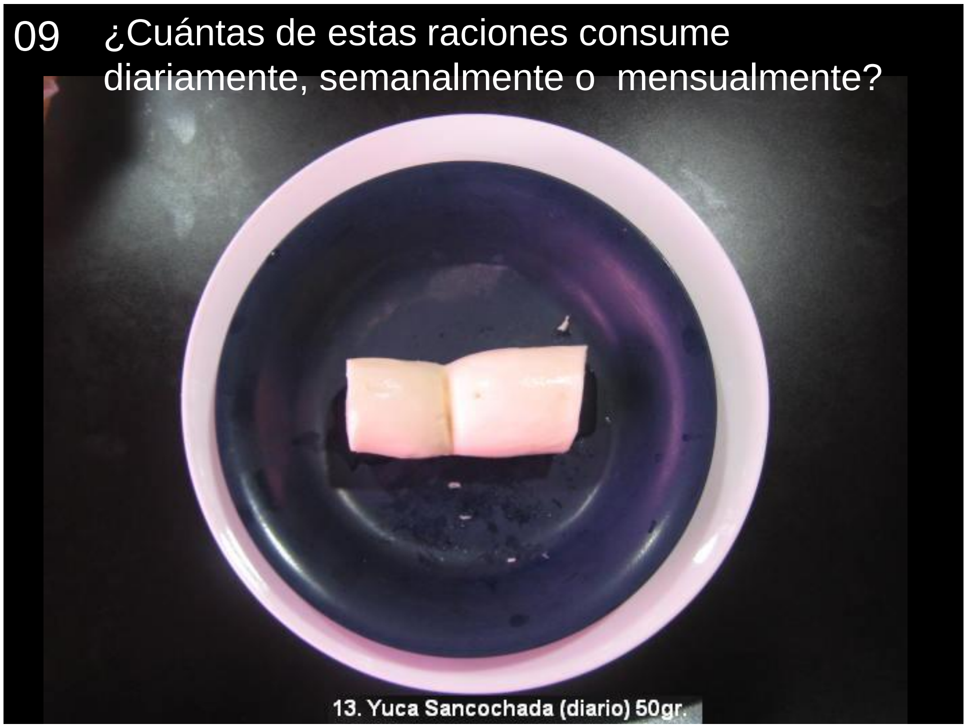
09
¿Cuántas de estas raciones consume diariamente, semanalmente o mensualmente?
13. Yuca Sancochada (diario) 50gr.

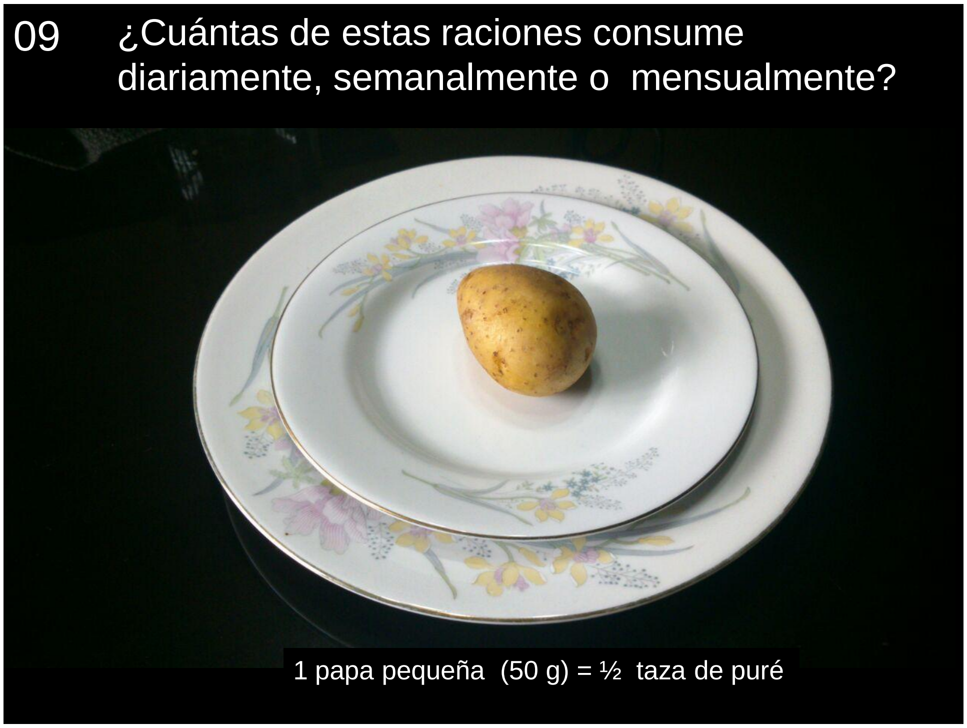
09
¿Cuántas de estas raciones consume diariamente, semanalmente o mensualmente?
1 papa pequeña (50 g) = ½ taza de puré

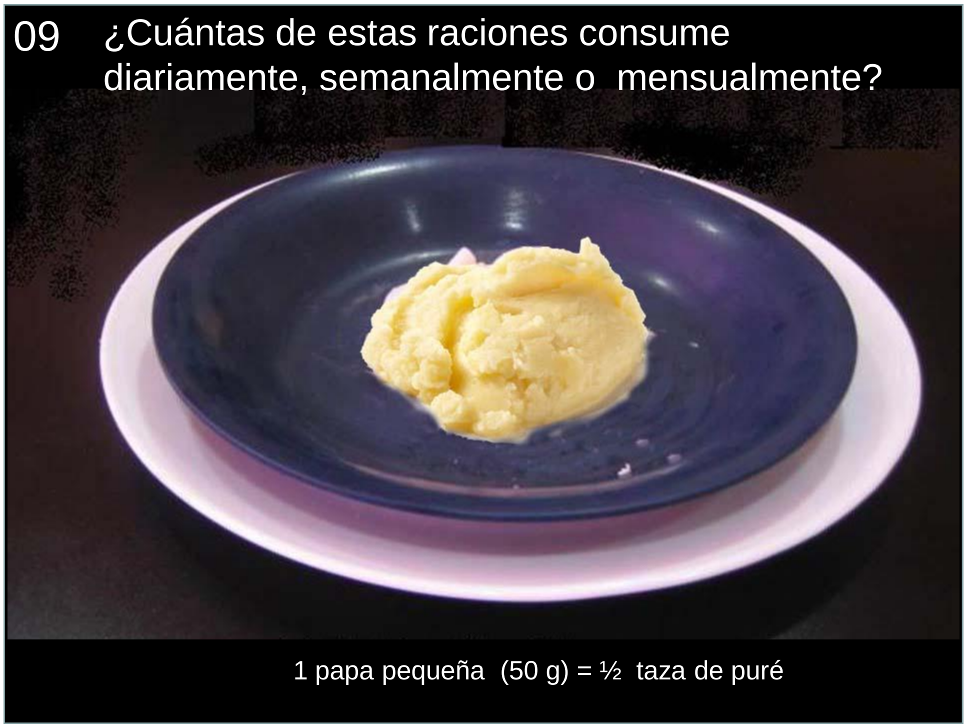
09
¿Cuántas de estas raciones consume diariamente, semanalmente o mensualmente?
1 papa pequeña (50 g) = ½ taza de puré

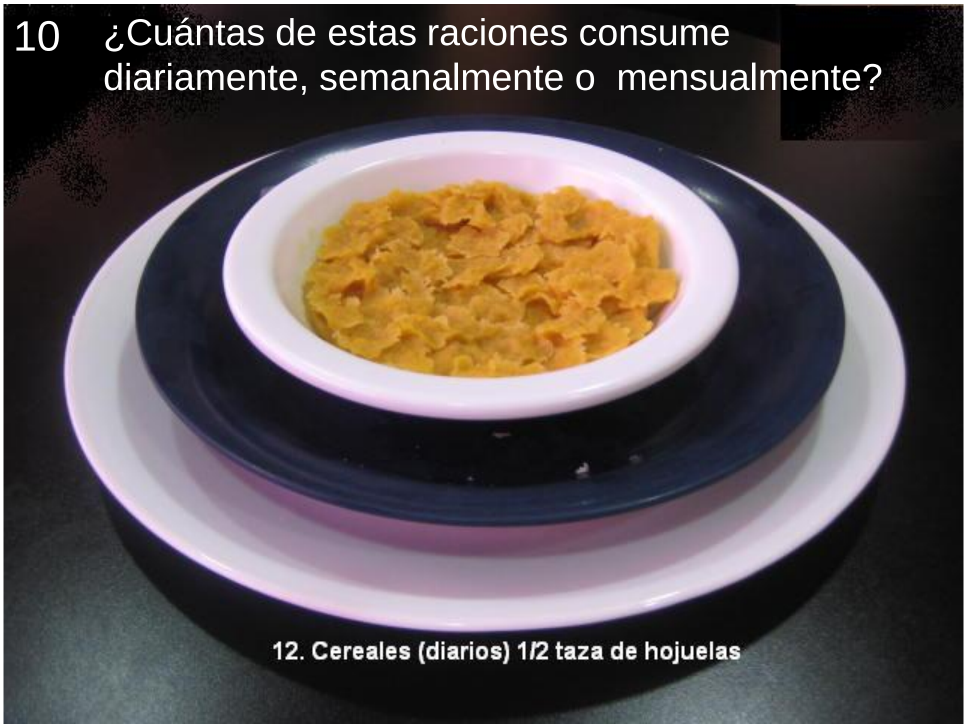
10
¿Cuántas de estas raciones consume diariamente, semanalmente o mensualmente?
12. Cereales (diarios) 1/2 taza de hojuelas

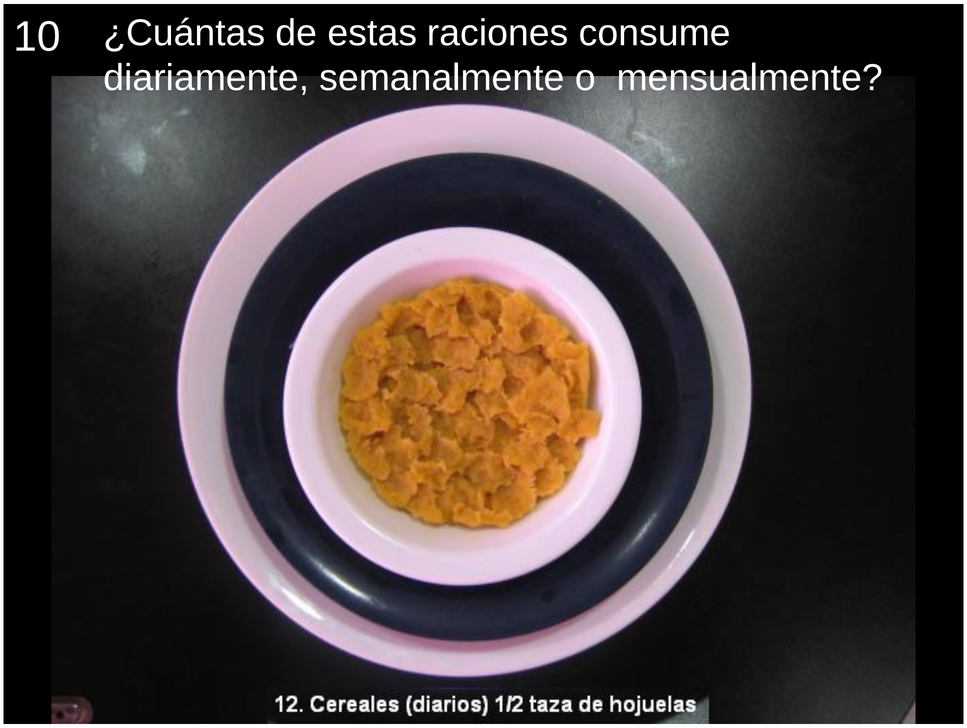
10
¿Cuántas de estas raciones consume diariamente, semanalmente o mensualmente?
12. Cereales (diarios) 1/2 taza de hojuelas

11 ¿Cuántas de estas raciones consume diariamente, semanalmente o mensualmente?

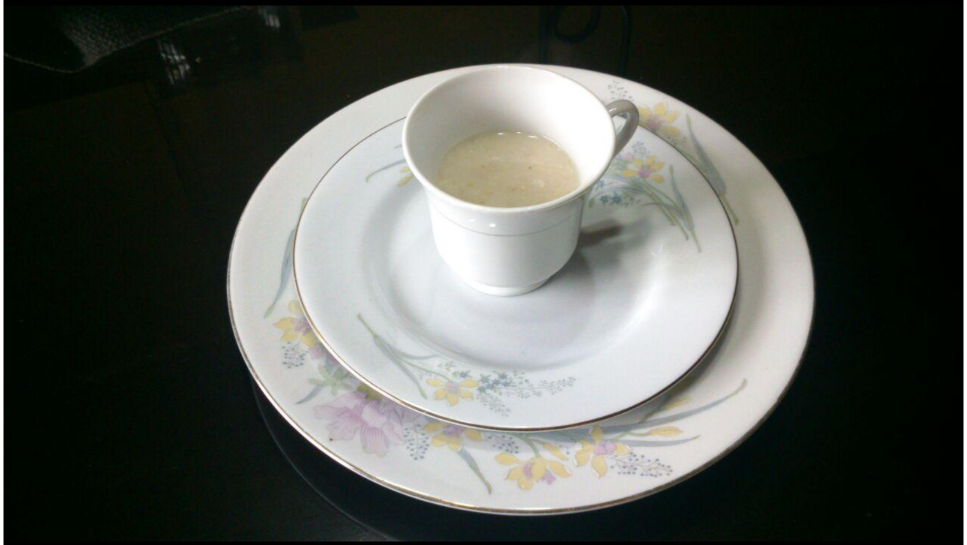

Avena en hojuelas preparada: ½ taza cocida

12 ¿Cuántas de estas raciones consume diariamente, semanalmente o mensualmente?

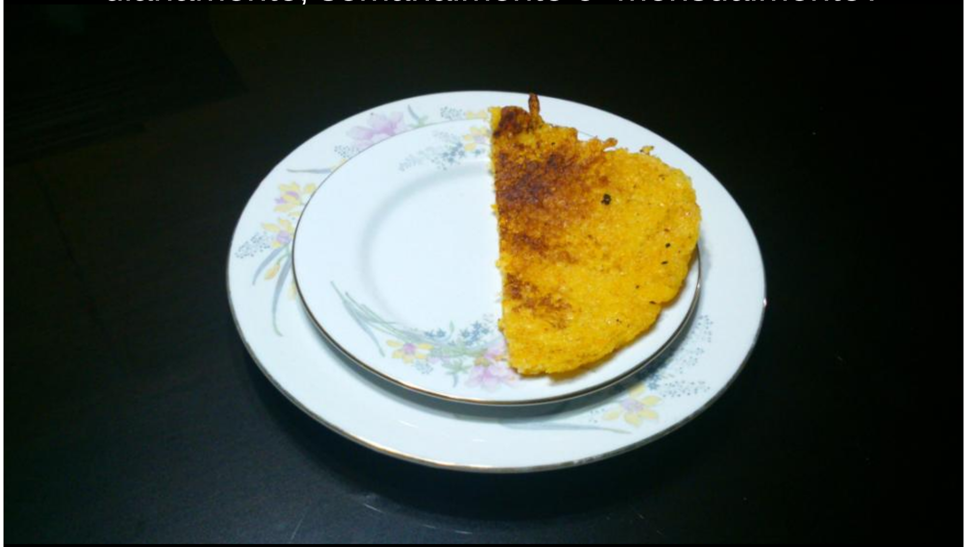

½ cachapa pequeña (80 g)

13 ¿Cuántas de estas raciones consume diariamente, semanalmente o mensualmente?

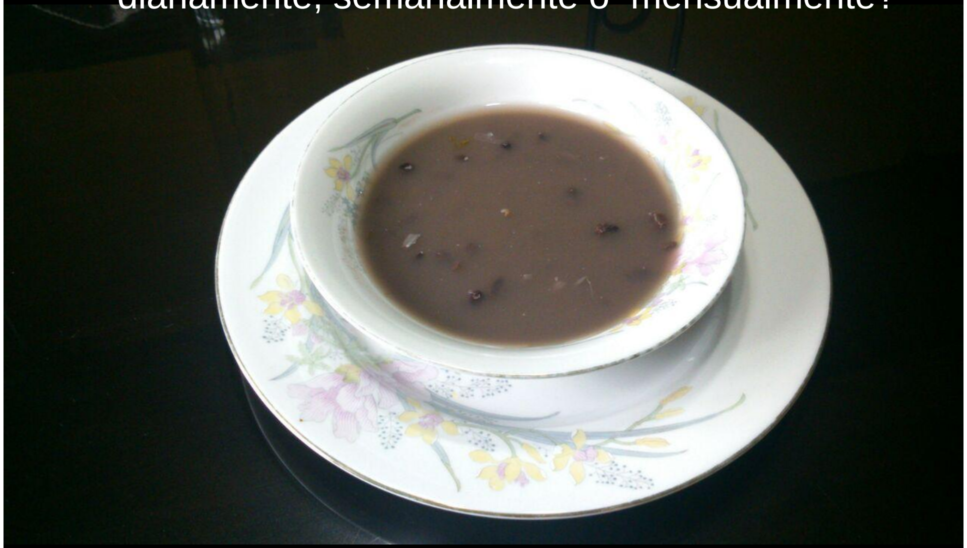

1 taza cocida (210 g)

14 ¿Cuántas de estas raciones consume diariamente, semanalmente o mensualmente?

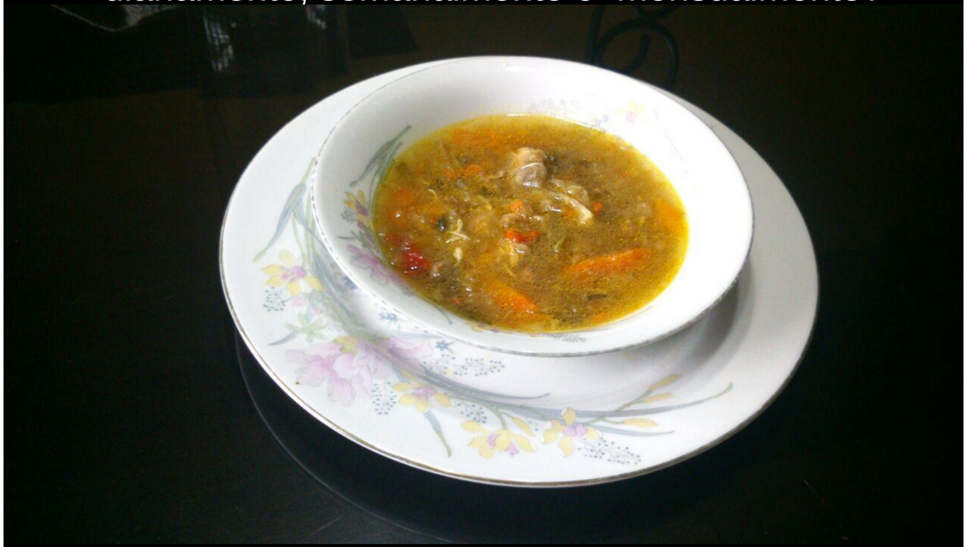

Sopa: 1 taza (240 g)

15 ¿Cuántas de estas raciones consume diariamente, semanalmente o mensualmente?

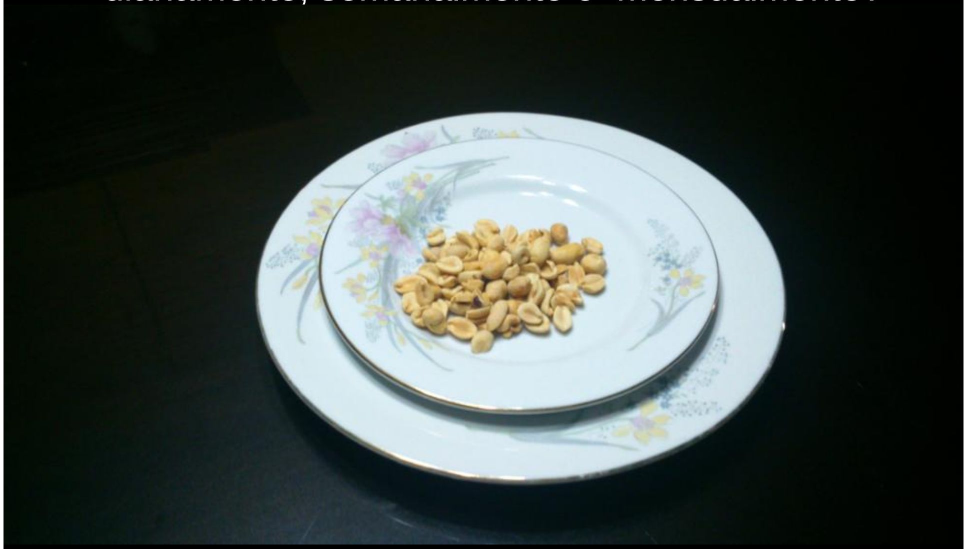

nueces, maní, pistachos, avellanas, almendras: ½ taza o 3 cucharadas (25 g)

16 ¿Cuántas de estas raciones consume diariamente, semanalmente o mensualmente?

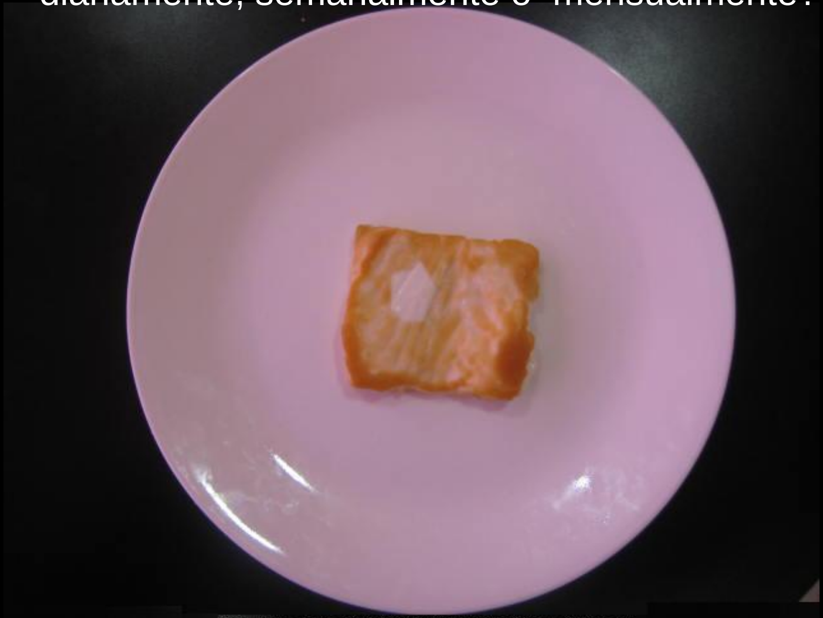

23. Pescado (diario) 1 filite mediano 120 gr.

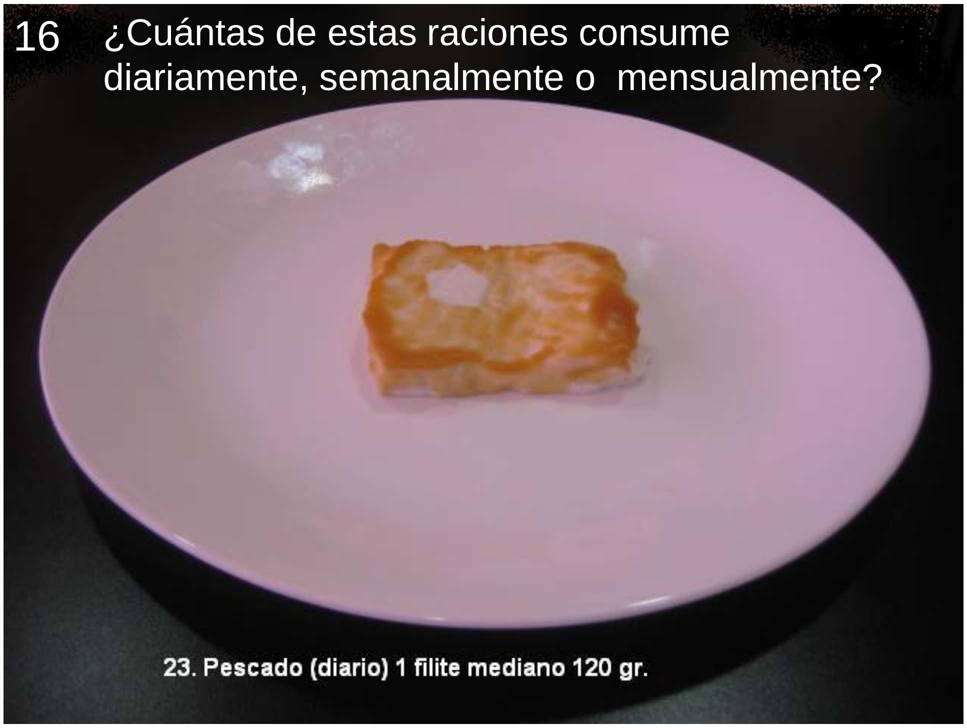
16
¿Cuántas de estas raciones consume diariamente, semanalmente o mensualmente?
23. Pescado (diario) 1 filite mediano 120 gr.

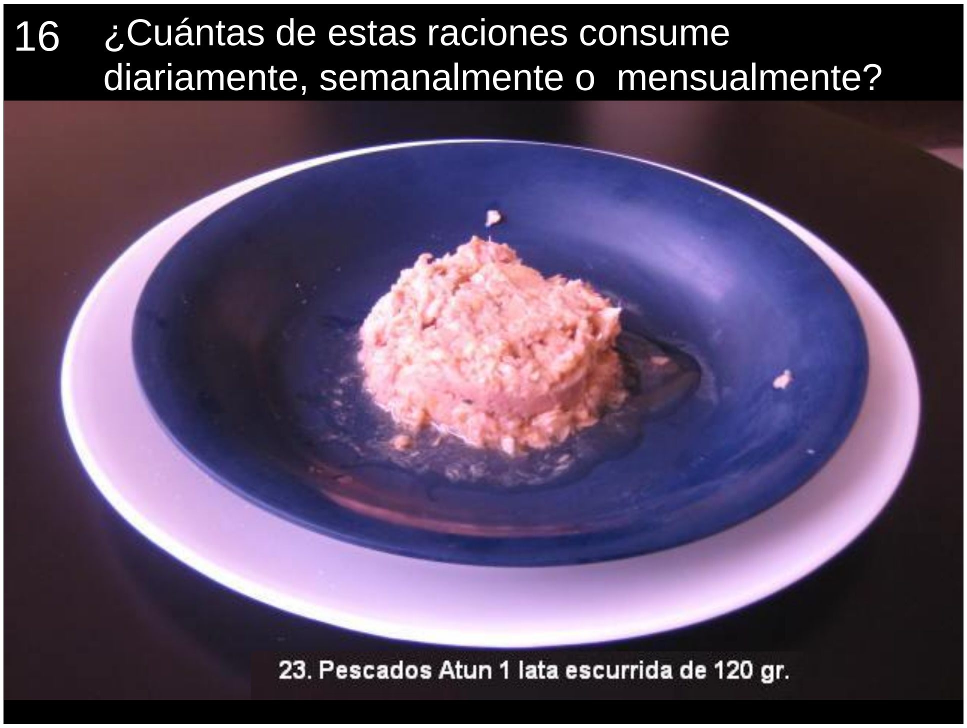
16
¿Cuántas de estas raciones consume diariamente, semanalmente o mensualmente?
23. Pescados Atun 1 lata escurrida de 120 gr.

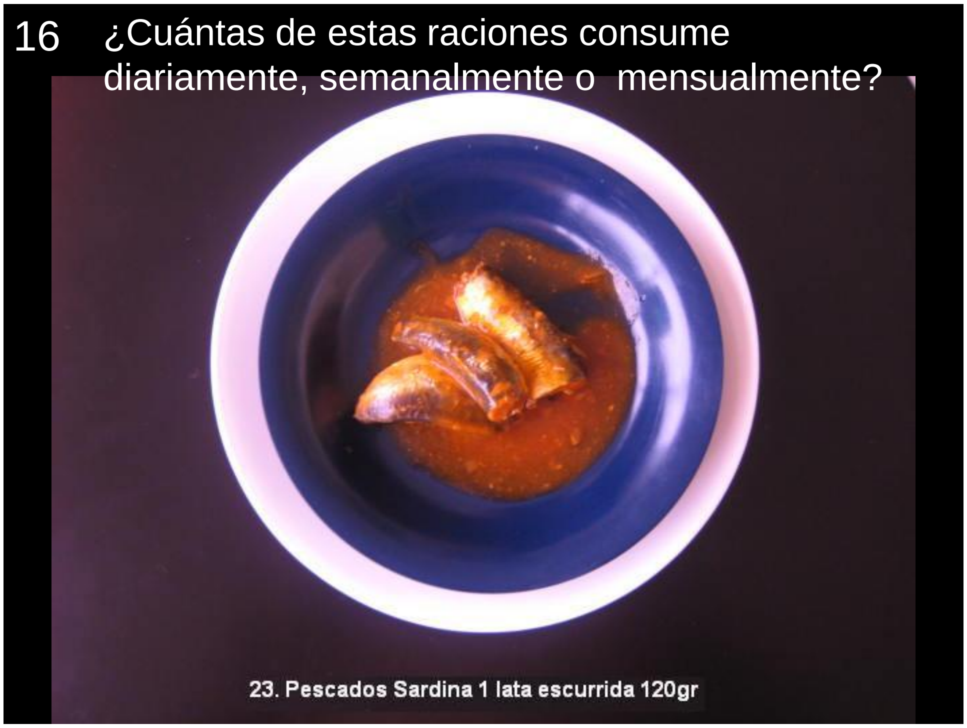
16
¿Cuántas de estas raciones consume diariamente, semanalmente o mensualmente?
23. Pescados Sardina 1 lata escurrida 120gr

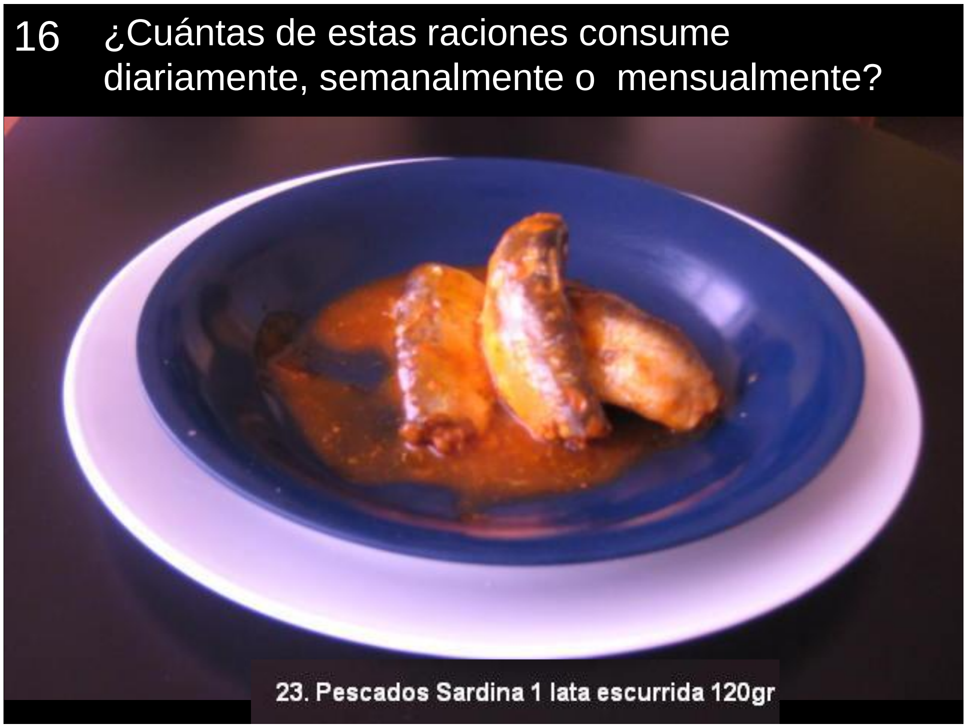
16
¿Cuántas de estas raciones consume diariamente, semanalmente o mensualmente?
23. Pescados Sardina 1 lata escurrida 120gr

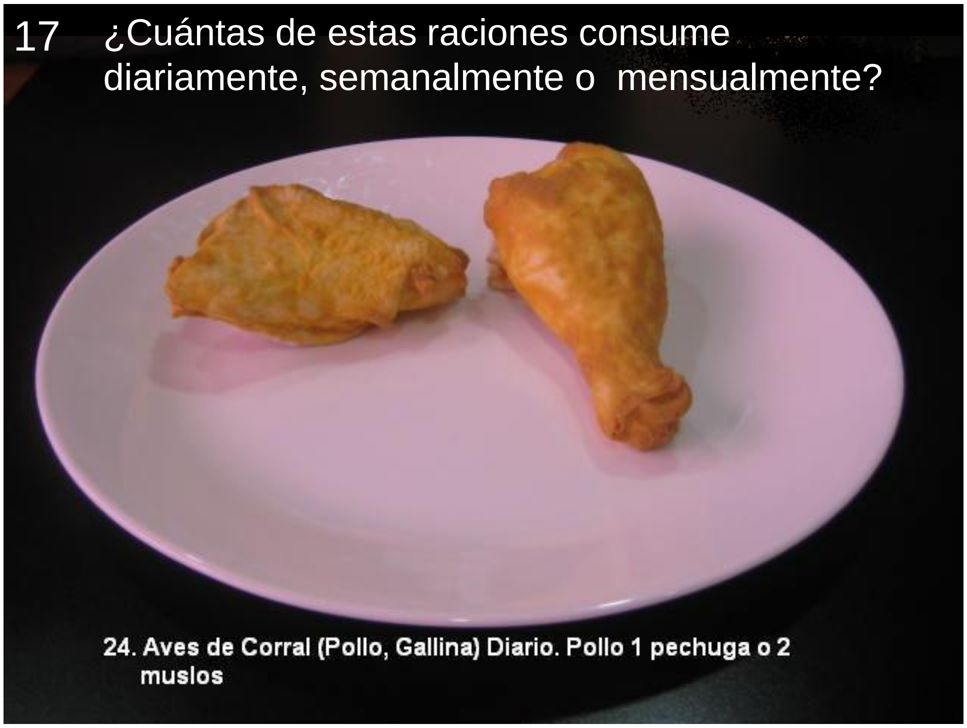
17
¿Cuántas de estas raciones consume diariamente, semanalmente o mensualmente?
24. Aves de Corral (Pollo, Gallina) Diario. Pollo 1 pechuga o 2 muslos

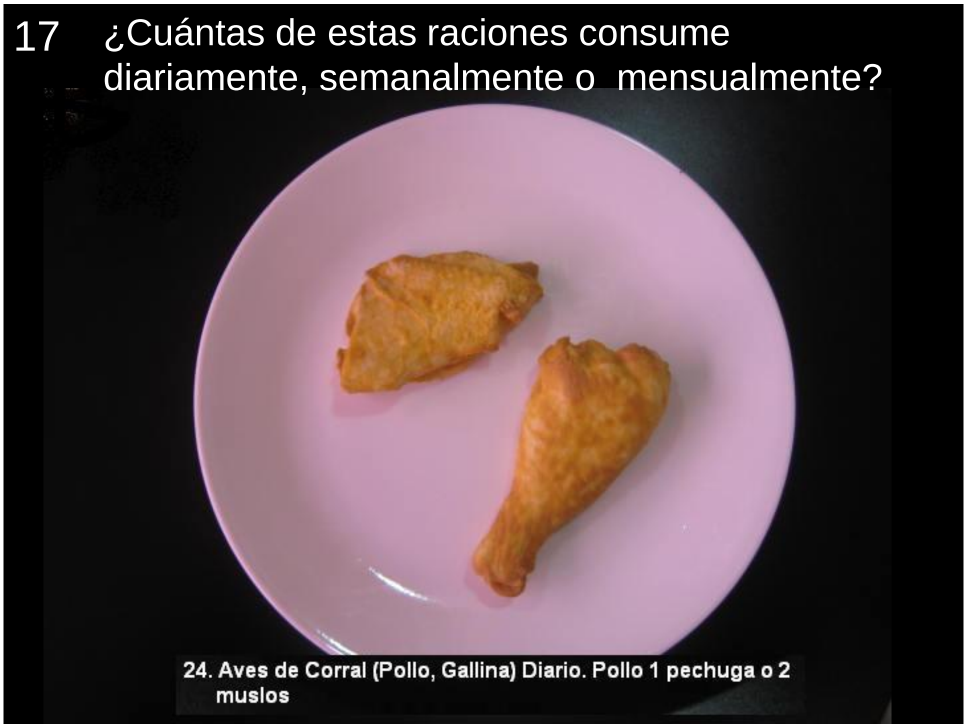
17
¿Cuántas de estas raciones consume diariamente, semanalmente o mensualmente?
24. Aves de Corral (Pollo, Gallina) Diario. Pollo 1 pechuga o 2 muslos

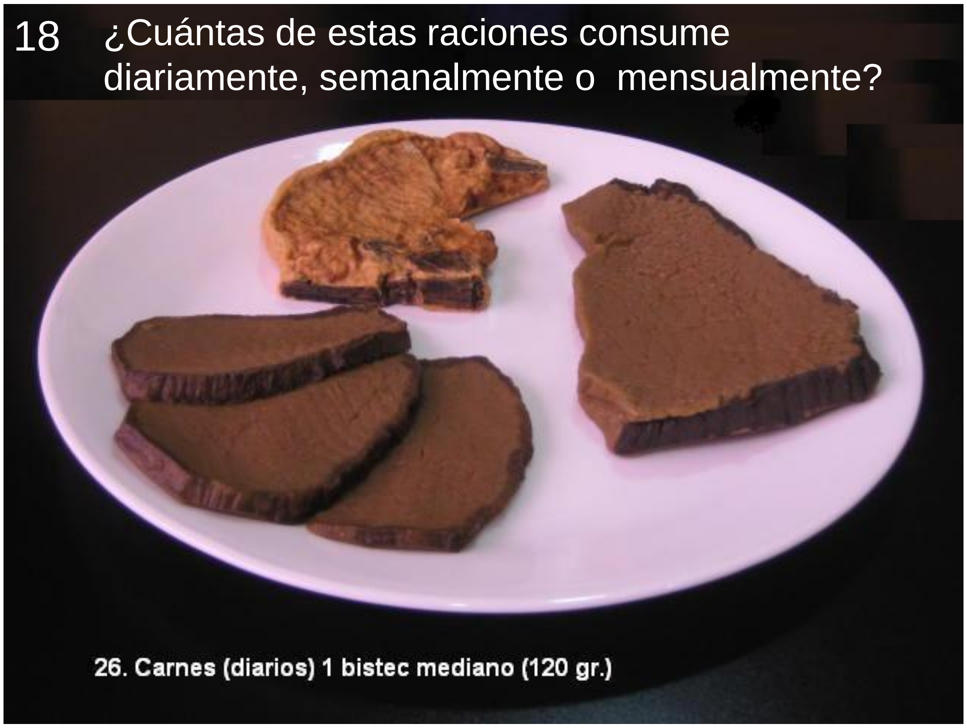
18
¿Cuántas de estas raciones consume diariamente, semanalmente o mensualmente?
26. Carnes (diarios) 1 bistec mediano (120 gr.)

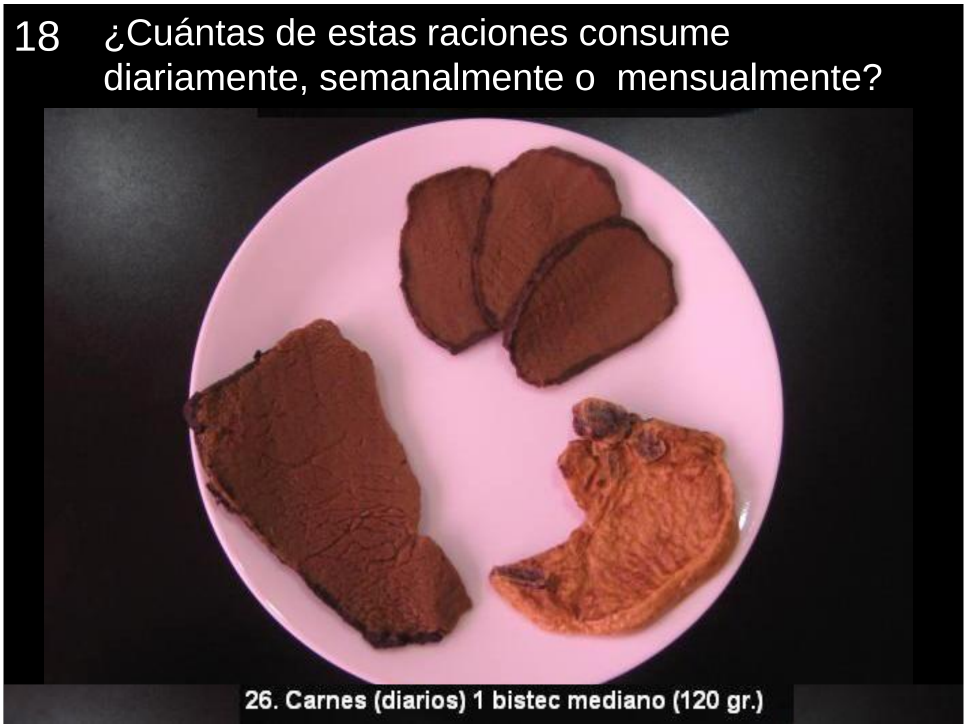
18
¿Cuántas de estas raciones consume diariamente, semanalmente o mensualmente?
26. Carnes (diarios) 1 bistec mediano (120 gr.)

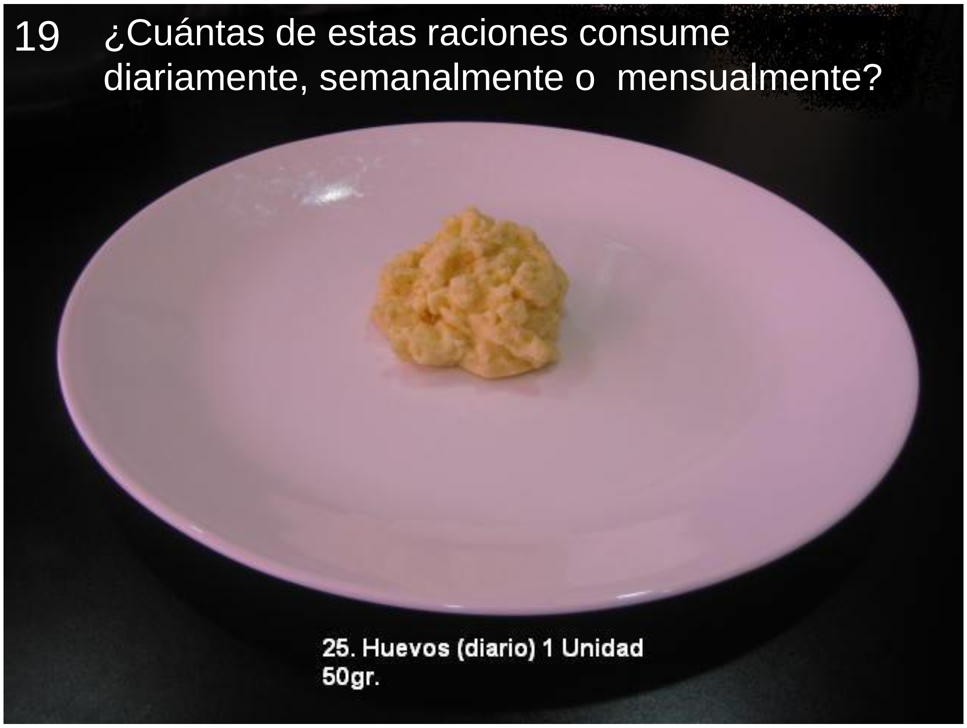
19
¿Cuántas de estas raciones consume diariamente, semanalmente o mensualmente?
25. Huevos (diario) 1 Unidad 50gr.

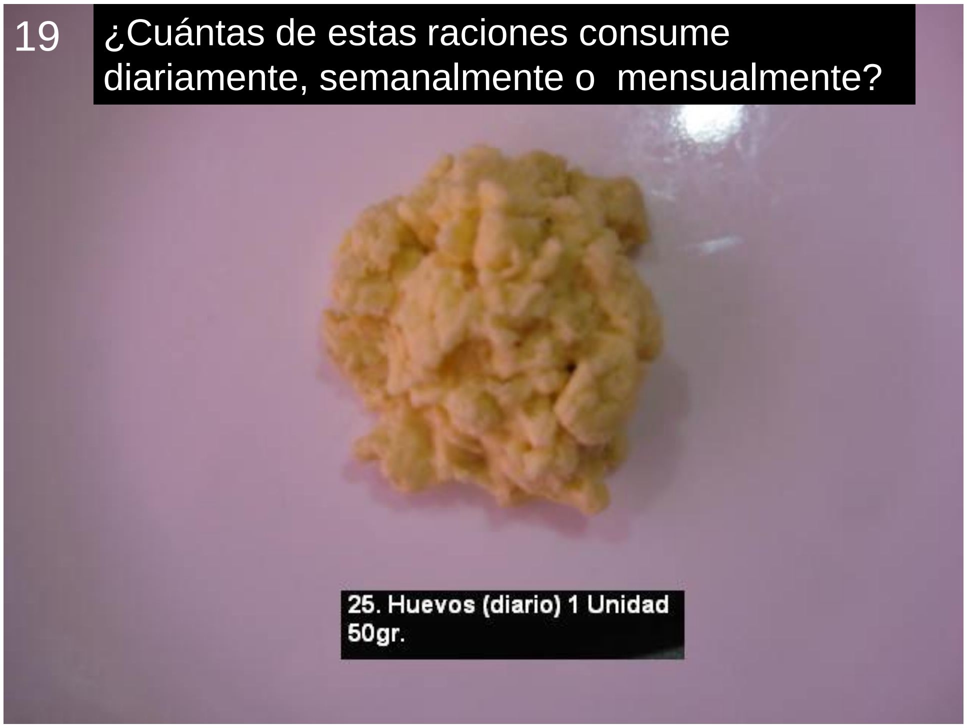
19
¿Cuántas de estas raciones consume diariamente, semanalmente o mensualmente?
25. Huevos (diario) 1 Unidad 50gr.

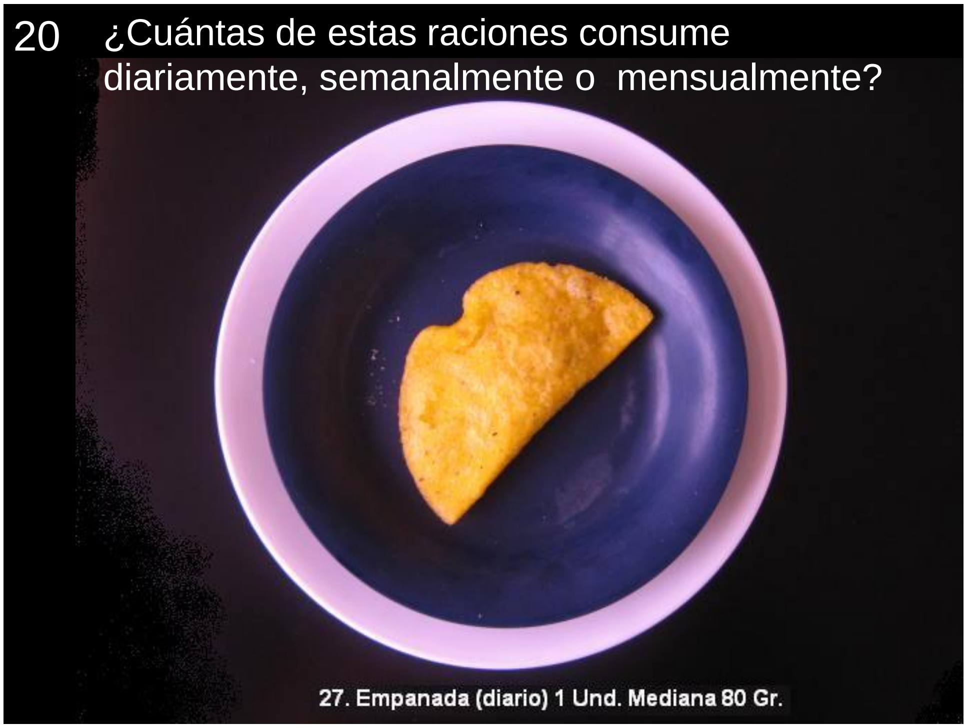
20
¿Cuántas de estas raciones consume diariamente, semanalmente o mensualmente?
27. Empanada (diario) 1 Und. Mediana 80 Gr.

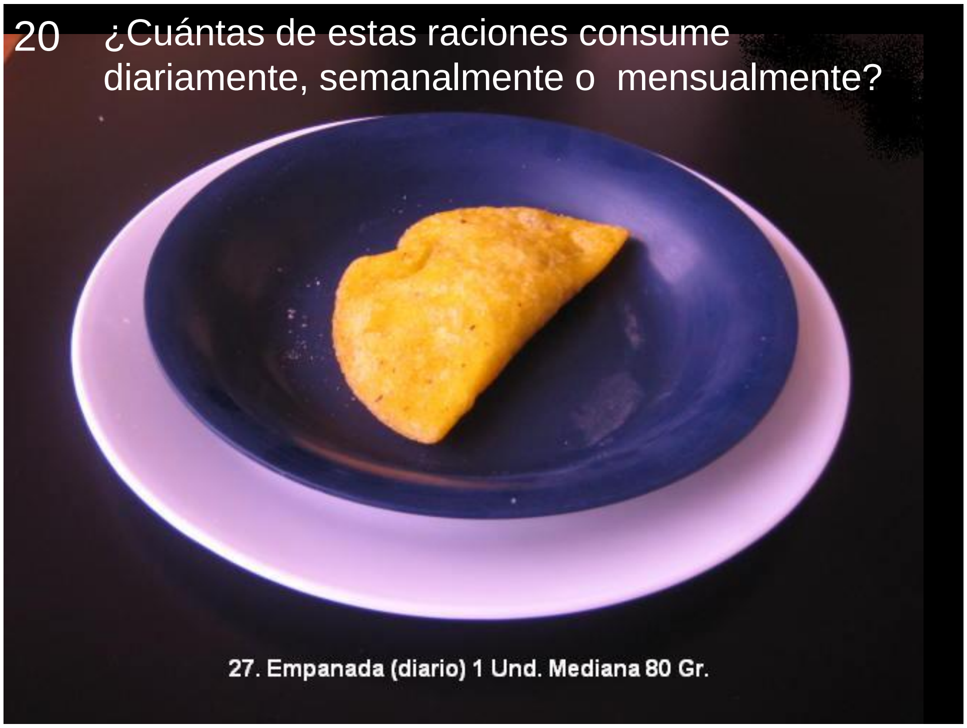
20
¿Cuántas de estas raciones consume diariamente, semanalmente o mensualmente?
27. Empanada (diario) 1 Und. Mediana 80 Gr.

21 ¿Cuántas de estas raciones consume diariamente, semanalmente o mensualmente?

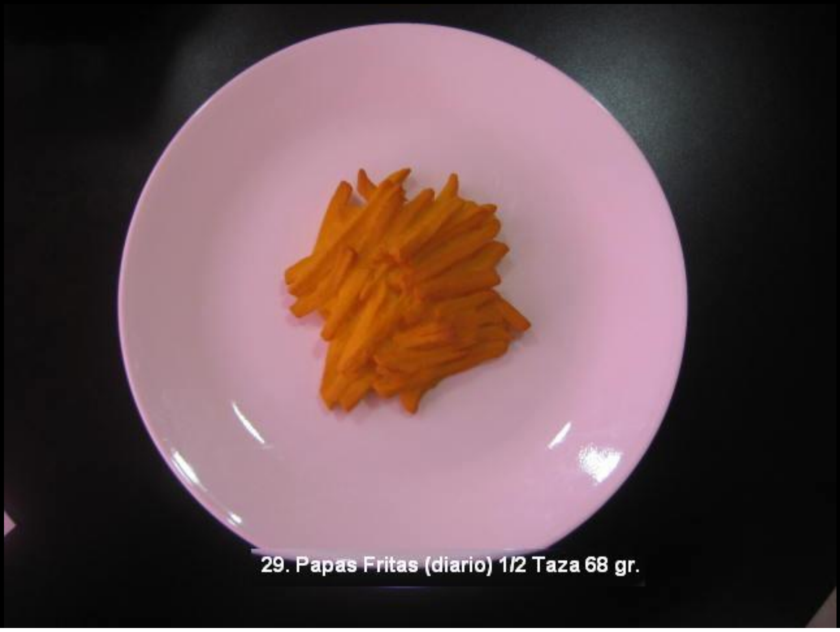

21 ¿Cuántas de estas raciones consume diariamente, semanalmente o mensualmente?

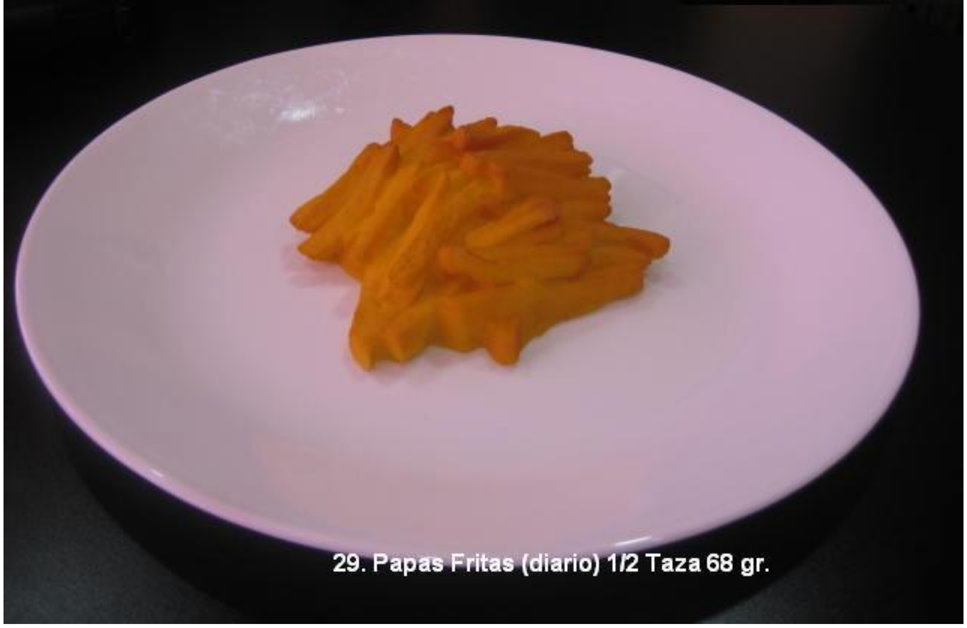

21 ¿Cuántas de estas raciones consume diariamente, semanalmente o mensualmente?

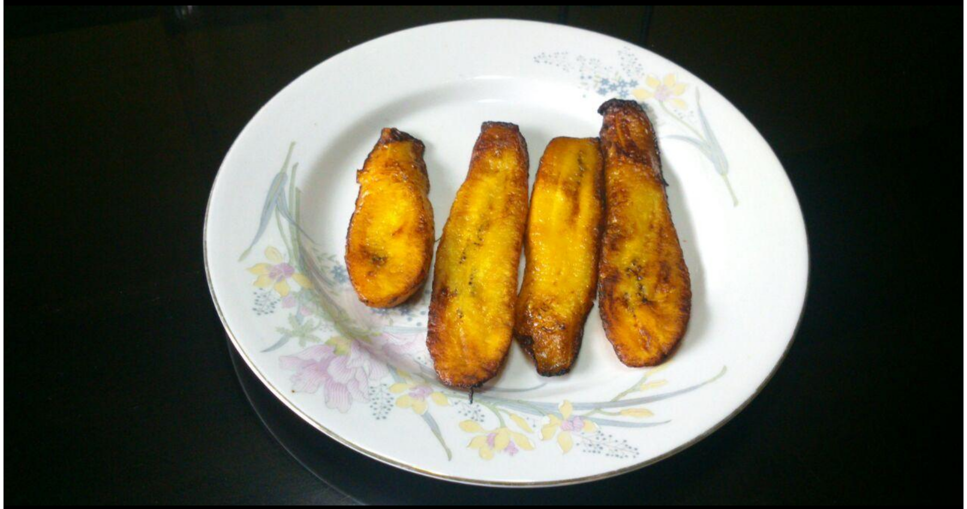

Tajada: ½ plátano = 4 unidades(150 g)

21 ¿Cuántas de estas raciones consume diariamente, semanalmente o mensualmente?

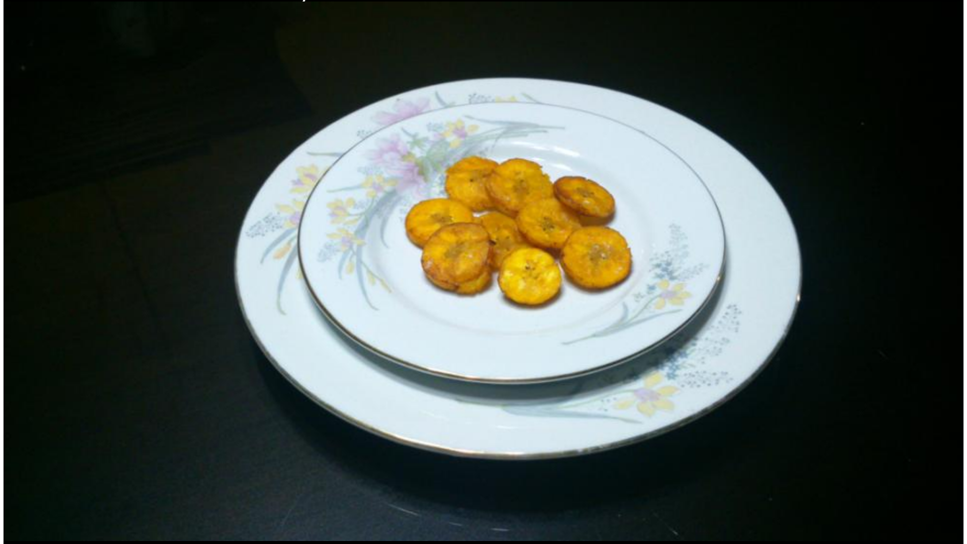

Tostones ½ plátano = 4 unidades (150 g)

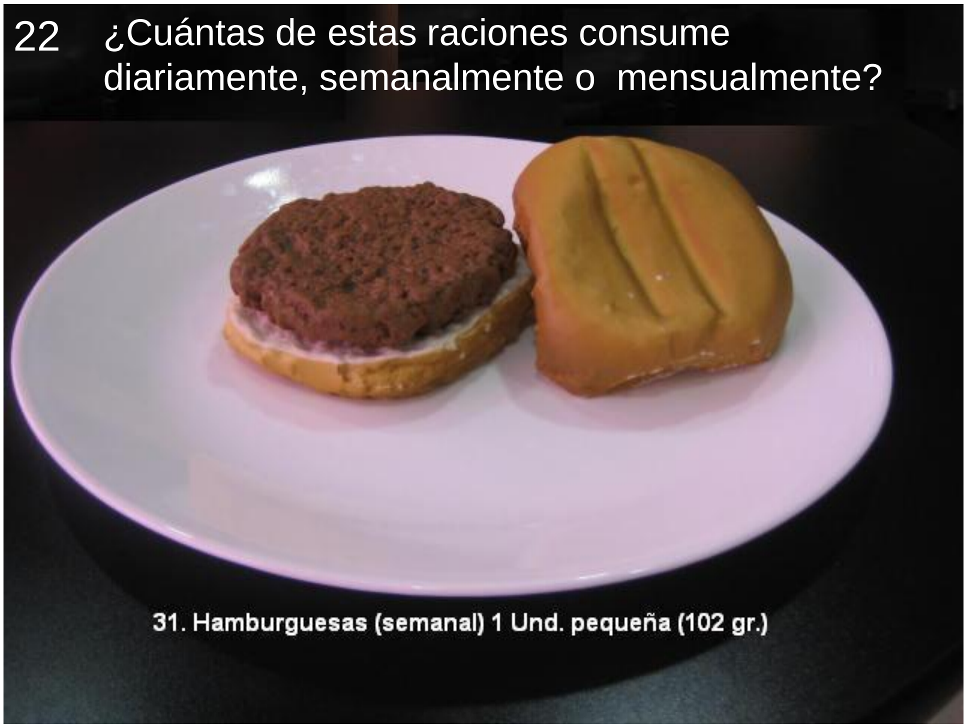
22
¿Cuántas de estas raciones consume diariamente, semanalmente o mensualmente?
31. Hamburguesas (semanal) 1 Und. pequeña (102 gr.)

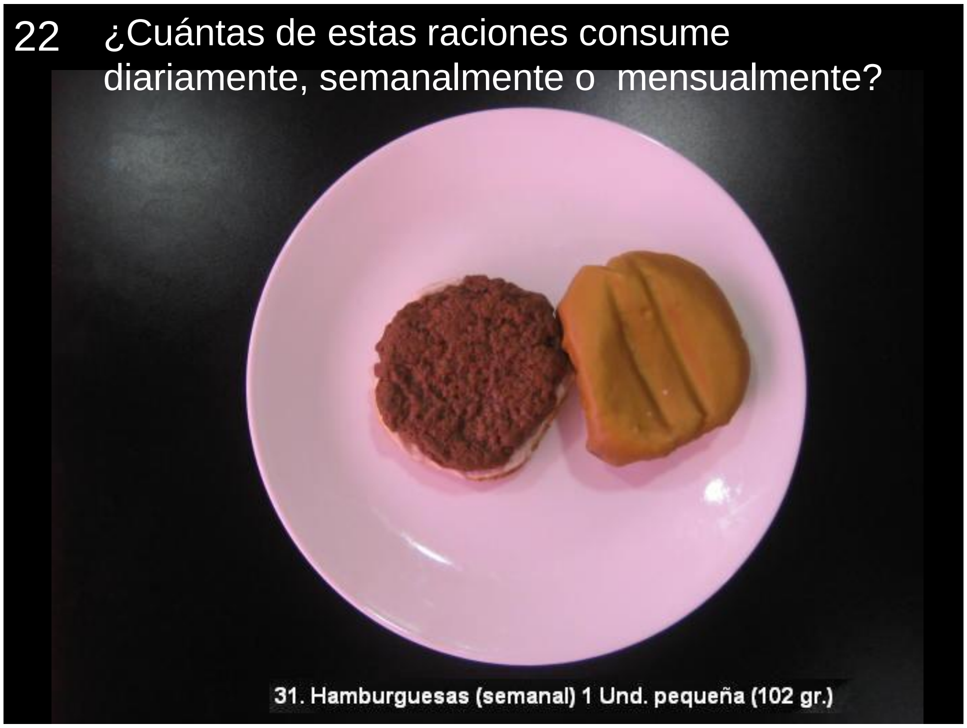
22
¿Cuántas de estas raciones consume diariamente, semanalmente o mensualmente?
31. Hamburguesas (semanal) 1 Und. pequeña (102 gr.)

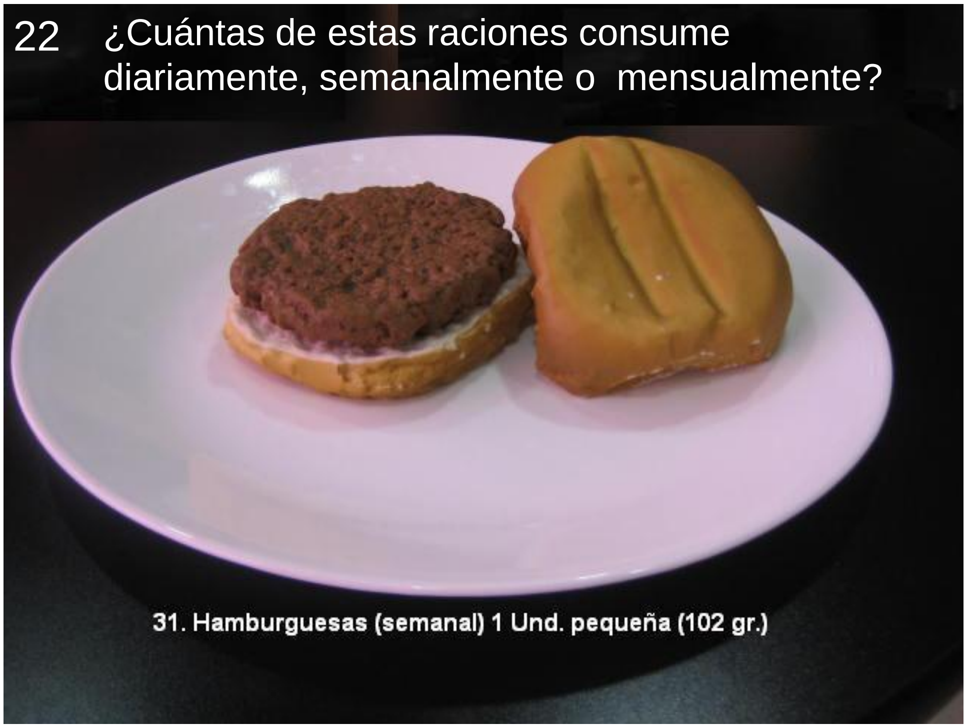
22
¿Cuántas de estas raciones consume diariamente, semanalmente o mensualmente?
31. Hamburguesas (semanal) 1 Und. pequeña (102 gr.)

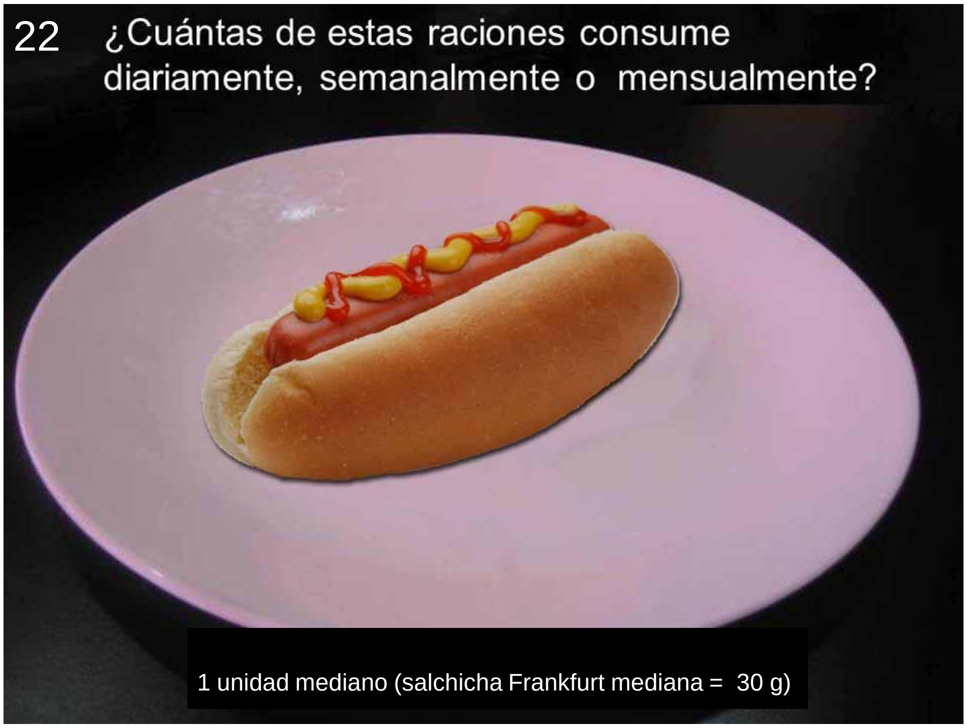
22
¿Cuántas de estas raciones consume diariamente, semanalmente o mensualmente?
1 unidad mediano (salchicha Frankfurt mediana = 30 g)

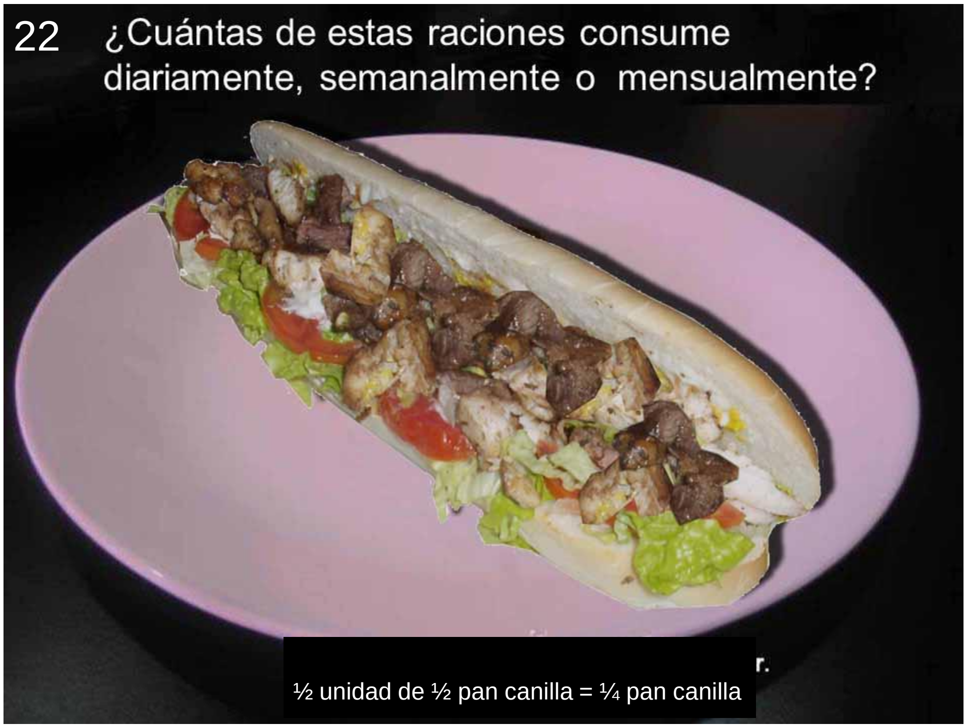
22
¿Cuántas de estas raciones consume diariamente, semanalmente o mensualmente?
½ unidad de ½ pan canilla = ¼ pan canilla

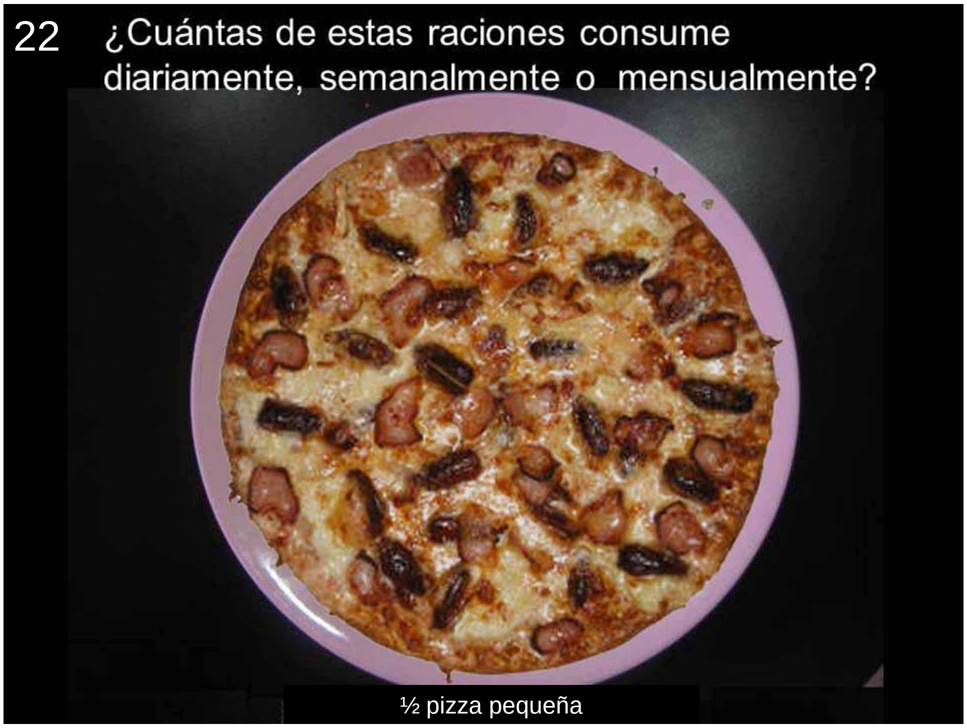
22
¿Cuántas de estas raciones consume diariamente, semanalmente o mensualmente?
½ pizza pequeña

23 ¿Cuántas de estas raciones consume diariamente, semanalmente o mensualmente?

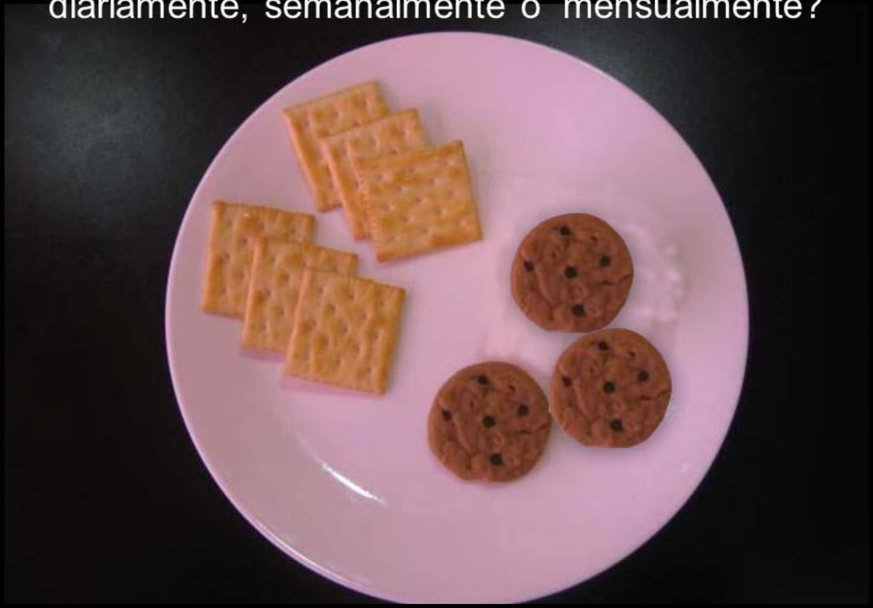

- Galleta dulce: 1 paquete (30 g) - Galleta de soda: 1 paquete

24 ¿Cuántas de estas raciones consume diariamente, semanalmente o mensualmente?

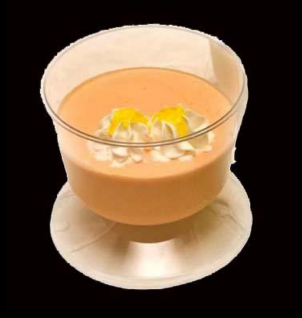

Dulces y postres: ½ taza = 1 onza (33 g)

25 ¿Cuántas de estas raciones consume diariamente, semanalmente o mensualmente?

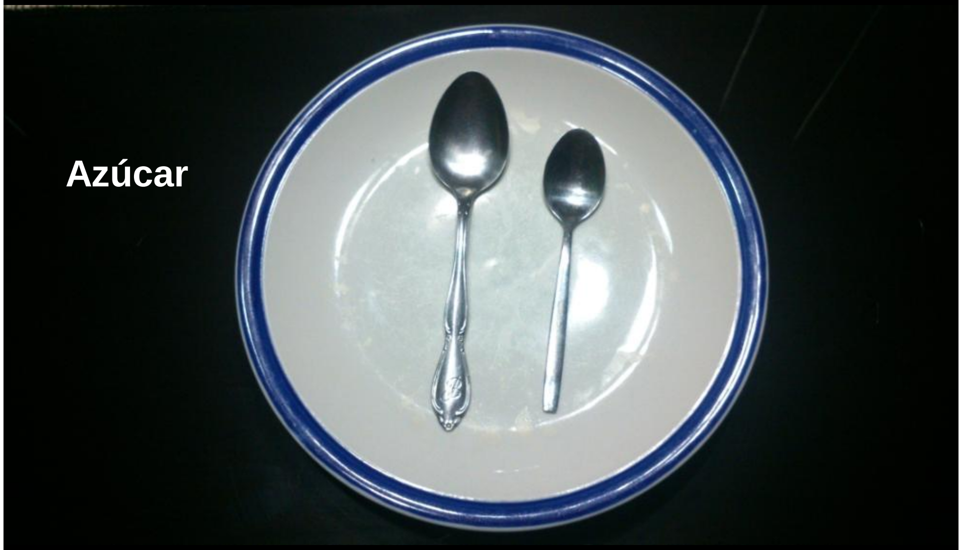

Azúcar: 1 cucharada = 15 g 1 cucharadita = 5 g

26 ¿Cuántas veces come usted en un establecimiento de comida rápida?

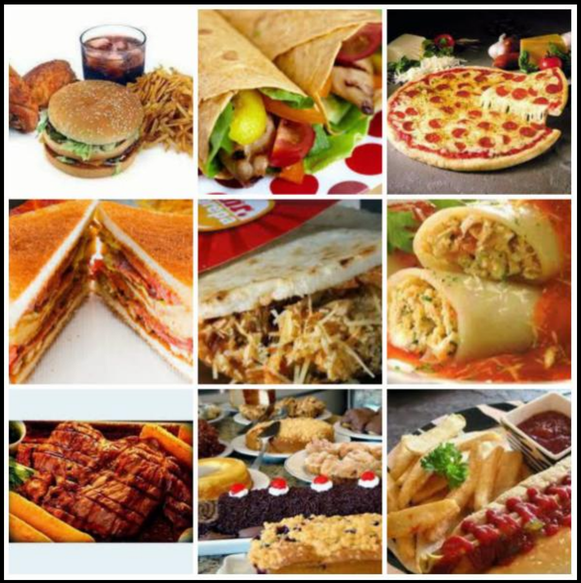

27 ¿Cuántas de estas raciones consume diariamente, semanalmente o mensualmente?

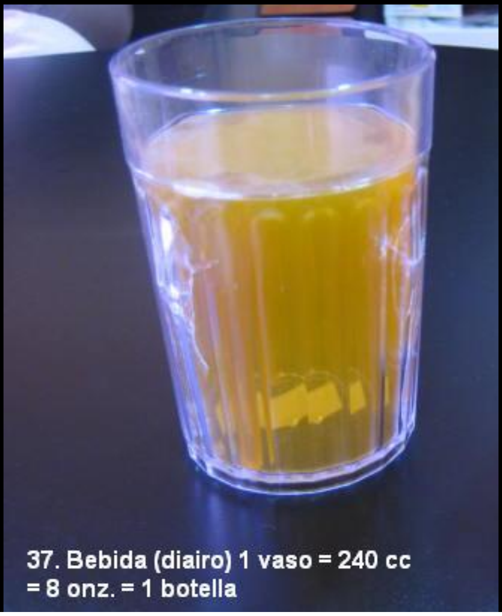

37. Bebida (diairo) 1 vaso = 240 cc = 8 onz. = 1 botella

28 ¿Cuántas de estas raciones consume diariamente, semanalmente o mensualmente?

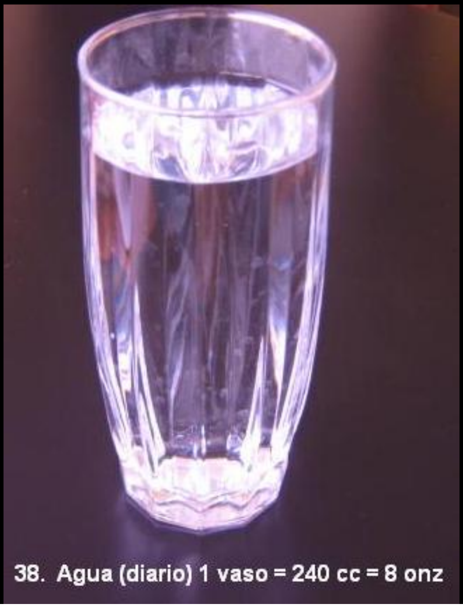

38. Agua (diario) 1 vaso = 240 cc = 8 onz

29 ¿Cuántas de estas raciones consume diariamente, semanalmente o mensualmente?

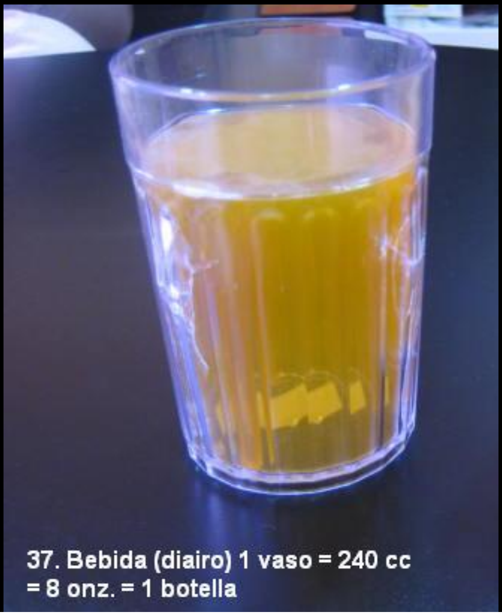

30 ¿Cuántas de estas raciones consume diariamente, semanalmente o mensualmente?

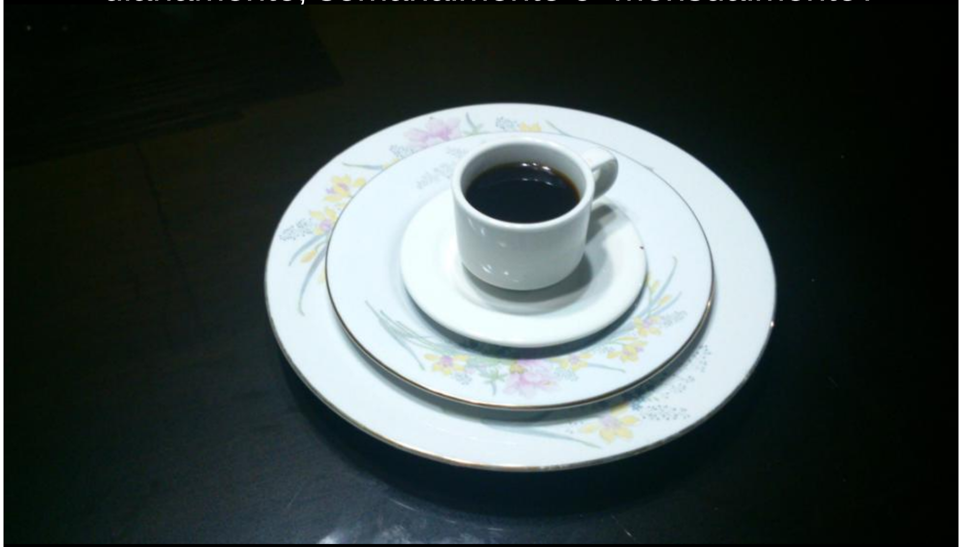

Café = 1 taza mediana
